# Neutrophils in Cancer: Phenotypic Heterogeneity Across Tumor Models and Significant Alteration of Splenic Neutrophil Phenotype in Lymphosarcoma RLS_40_ Model Following DNase I Treatment

**DOI:** 10.3390/cancers17162631

**Published:** 2025-08-12

**Authors:** Khetam Sounbuli, Ludmila A. Alekseeva, Aleksandra V. Sen’kova, Oleg V. Markov, Innokenty A. Savin, Marina A. Zenkova, Nadezhda L. Mironova

**Affiliations:** 1 Institute of Chemical Biology and Fundamental Medicine SB RAS, Lavrentiev Ave., 8, Novosibirsk 630090, Russia; khetam.sounbuli.edu@gmail.com (K.S.); alekseeva@1bio.ru (L.A.A.); senkova_av@niboch.nsc.ru (A.V.S.); markov_oleg@list.ru (O.V.M.); savin_ia@niboch.nsc.ru (I.A.S.); marzen@niboch.nsc.ru (M.A.Z.); 2 Faculty of Natural Sciences, Novosibirsk State University, Pirogova st., 1, Novosibirsk 630090, Russia

**Keywords:** neutrophil, tumor-associated neutrophils, splenic neutrophils, neutrophil extracellular traps, NETosis, DNase I

## Abstract

**Background/Objectives**: Neutrophils have recently gained significant attention due to their heterogeneity in tumor settings. Recent data showed neutrophil pro- and anti-tumor profiles during tumor progression. However, the concessive causes of neutrophil skewing toward one or another profile are not fully understood. **Methods**: In this study, using RT-qPCR, flowcytometry, and confocal microscopy, we investigated the phenotype of splenic neutrophils of mice bearing Lewis lung carcinoma LLC, RLS_40_ lymphosarcoma, and B16 melanoma. **Results**: Our data showed an immunosuppressive phenotype in the case of the LLC model with PD-L1 and IL10 expression. In the B16 model, minimal changes in the neutrophil phenotype were observed, regardless of tumor size. In the RLS_40_ model, the neutrophil phenotype was associated with the tumor growth rate, where, in aggressively progressed tumors (RLS_40_^High^), CCL17 was expressed, while, in mice with controlled tumor growth (RLS_40_^Low^), anti-tumor markers were expressed (FAS, ICAM-1, PD-L1). DNase I treatment significantly reduced tumor growth and metastasis in the RLS_40_ model but not in B16, enhanced the anti-tumor profile in RLS_40_ neutrophils, and tended to reduce NET formation induced by A23187. **Conclusions**: The phenotype of neutrophils from tumor-bearing mice is influenced by the tumor type and progression stage. DNase I had anti-tumor, antimetastatic, and immunostimulatory effects and significantly modified the neutrophil profile in the immunogenic model RLS_40_.

## Simple Summary

Neutrophils, key cells of the innate immune system, have recently been identified as major players in tumor immunity. Recent data have shown their pro- and anti-tumor roles within the tumor microenvironment; however, it is not fully understood how neutrophils are directed to play one role or the other. To take the first step toward understanding the factors influencing neutrophil heterogeneity during tumor development, we studied the expression of main pro- and anti-tumor markers in splenic neutrophils from mice bearing tumors of different origins that cause distinct immune responses. We then applied a novel anti-tumor treatment, DNase I, and examined the resulting changes in the expression of these markers. The results of our study enhance the understanding of the role of neutrophils in fighting tumors and suggest novel anti-tumor therapies focused on neutrophil function modulation.

## 1. Introduction

Neutrophils are the key cells of the innate immune system and the first line of defense against pathogens. They represent the most abundant leukocyte type in human blood and the second most abundant type in murine blood [1]. Neutrophils have gained much attention in cancer studies [2]. The recently revealed heterogeneity of neutrophils in cancer raised the interest to study the alteration of neutrophil phenotypes in different tumor models [3]. It has been shown that two phenotypes of tumor-associated neutrophils (TANs) can be found in tumors: N1 (anti-tumor) and N2 (pro-tumor). The pro- or anti-tumor functions of TANs seem to depend on the specific tumor microenvironment and the interplay between tumor cells and TANs [4]. Neutrophil polarization is a complex process influenced by a number of tumor-related factors. Currently, contradictory data are accumulating about the impact of neutrophils in tumor development and progression [5,6]. The dual role of neutrophils in tumor development may partially explain the fact that, in cancer patients, the phenotype of tumor-associated neutrophils determines a worse or favorable disease outcome [7,8]. Thus, the study of the phenotype and functioning of neutrophils in tumor development is relevant.

Recently, researchers have begun to pay more attention to neutrophil populations in lymphoid organs. It was shown that neutrophils migrate to the lymph nodes at the early stages of cancer progression and activate T cell proliferation, thereby maintaining adaptive anti-cancer immunity [9]. Splenic neutrophils have also gained more attention in the last few years. Although the spleen is a lymphoid organ, neutrophils accumulate in the spleen and are believed to play a vital role in different physiological and pathological conditions [10,11]. In tumor-bearing mice, splenic neutrophils could characterize the immune status of the organism and represent TANs or their precursors [12,13,14,15]. In contrast to bone-marrow-derived neutrophils, splenic neutrophils are characterized by a relatively high maturation state with preserved functionality [16,17,18]. Altogether, splenic neutrophils are a good representation of the immune response in the organism in different pathologies, especially in cancer.

TANs are capable of forming neutrophil extracellular traps (NETs) in the tumor microenvironment [19]. NETs are fibrinous structures composed of neutrophil genetic material and decorated with nuclear proteins and granule components [20] forming during the specific death of neutrophils, called NETosis [21]. NETs promote metastasis and tumor growth [19,22,23,24,25,26], protect the tumor and circulating tumor cells from the immune system [27,28,29], and also destroy the extracellular matrix and promote the formation of a premetastatic niche [30,31].

Numerous studies have shown that the level of cell-free DNA (cfDNA) in the blood of cancer patients is elevated, including an increased release of NETs by neutrophils [32,33,34]. Increased levels of cfDNA and NETs are often accompanied by a decrease in DNase activity in the blood [34,35]. Recent studies have identified NETs, cfDNA concentration, and blood DNase activity as important biomarkers of cancer development [36,37,38,39,40,41]. We proved the anti-tumor and antimetastatic effect of pancreatic bovine DNase I in different murine tumor models [42,43,44,45,46,47], which was also confirmed by others [48,49,50,51,52], making DNase I treatment a perspective direction in cancer therapy [53,54,55]. The mechanism underlying the anti-tumorigenic effects of DNase I could be explained by destroying tumorigenic DNA sequences or tumor-supporting NETs [47]. However, a more in-depth insight into the mechanism of DNase action could be associated with the immunomodulatory effect and the restoration of anti-tumor immunity [43,44,56].

In this study, we investigated the phenotype of splenic neutrophils of mice bearing LLC, RLS_40_, and B16 depending on the aggressiveness of tumor growth and the alterations of phenotype and functionality of splenic neutrophils with a decrease in the tumor invasive potential by bovine pancreatic DNase I.

## 2. Materials and Methods

### 2.1. Cell Cultures

The B16-F10 cell line was purchased from the Institute of Cytology of the Russian Academy of Sciences (St. Petersburg, Russia). The B16 cell line was cultured in DMEM (Dulbecco’s Modified Eagle Medium; Thermo Fisher Scientific, Waltham, MA, USA) and supplemented with 10% fetal bovine serum (FBS; HyClone, Logan, UT, USA) and 1% antibiotic-antimycotic solution (10 mg/mL streptomycin, 10,000 U/mL penicillin, and 25 μg/mL amphotericin (MP Biomedicals, Santa Ana, CA, USA)) at 37 °C in a humidified atmosphere containing 5% CO_2_ (standard conditions) and regular passages to maintain exponential growth.

Mouse RLS_40_ cells were obtained from the cell collection of the Institute of Chemical Biology and Fundamental Medicine (SB RAS) [47]. The RLS_40_ cells were cultured in IMDM supplemented with 10% FBS and 1% antibiotic-antimycotic solution under standard conditions in the presence of 40 μM vinblastine to maintain the MDR-phenotype.

### 2.2. Tumor Strains

The LLC tumor strain is permanently maintained in C57Bl mice in the form of a solid tumor in the vivarium of the Institute of Chemical Biology and Fundamental Medicine SB RAS. LLC cells were obtained from LLC solid node by tumor dissection followed by homogenization in PBS, passing through a 70 μm cell strainer (Corning, Glendale, AZ, USA) and centrifugation on a layer of the lymphocyte separation medium (MP Biomedicals, Santa Ana, CA, USA) at 300 g for 10 min. The cell pellet was washed with PBS and used immediately for implantation into the right thighs of the mice.

### 2.3. Mice

C57Bl/6 and CBA/LacSto (hereinafter C57Bl and CBA) male mice were obtained from the vivarium of the Institute of Chemical Biology and Fundamental Medicine SB RAS (Novosibirsk, Russia). The mice were housed in normal daylight conditions in plastic cages with an ad libitum supply of water and food. C57Bl/6 male mice, aged 3–4 months and weighing 20–25 g, were used for the B16 model; C57Bl/6 male mice, aged 4–5 months and weighing 25–30 g, were used for the LLC model; and CBA/LacSto mice, aged 3–4 months and weighing 25–30 g, were used for the RLS_40_ model.

All animal procedures were conducted in strict compliance with the guidelines for the proper use and care of laboratory animals (ECC Directive 2010/63/EU). The experimental protocols were approved by the Ethics Committee for Animal Experiments of the Institute of Cytology and Genetics SB RAS (Novosibirsk, Russia) (Protocol No. 49, 23 May 2019 and No. 188, 3 October 2024).

### 2.4. Tumor Implantation

**Figure 1:**
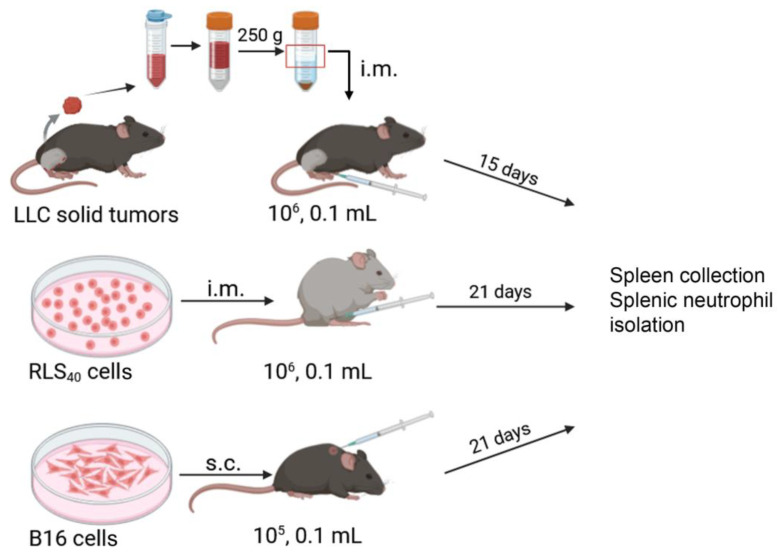
Design of animal experiment. LLC cells prepared from LLC solid node were implanted intramuscularly (10^6^ cells, in 0.1 mL PBS) into C57Bl mice. RLS_40_ (10^6^ cells, in 0.1 mL PBS) were implanted intramuscularly into CBA mice. B16 (10^5^ cells, in 0.1 mL PBS) were implanted subcutaneously into C57Bl/6 mice. Tumor sizes were measured on day 15 for LLC and day 21 for B16 and RLS_40_ tumor development, spleens were collected, and neutrophils were isolated from the spleens by magnetic positive selection.

*LLC with a primary tumor node*. The LLC strain was routinely propagated in vivo as described above. Then, LLC cells at the upper interface of the lymphocyte separation medium were collected, washed with PBS, and injected intramuscularly (10^6^ cells/mouse, i.m.) into the right thighs of mice (n = 15) (Figure 1).

*B16 with a primary tumor node.* B16 cells were grown in 25 cm^2^ cell culture flasks in DMEM medium supplemented with 10% FBS and antibiotic–antimycotic solution until ~90% confluence was reached (up to 48 h). The medium was discarded, and the cells were detached from the flask by trypsin, collected by centrifugation at 300× *g* for 5 min, washed twice with PBS, and immediately used for implantation. The model B16 was induced by subcutaneous (s.c.) implantation of B16 cells (10^5^ cells/mouse) in 0.1 mL of PBS in the withers of C57Bl mice (n = 15) (Figure 1).

*RLS_40_ with a primary tumor node.* The RLS_40_ cells were cultured in IMDM supplemented with 10% FBS and antibiotic–antimycotic solution as described above. The cells were collected by centrifugation at 300× *g* for 5 min, washed with PBS, and immediately used for implantation. Tumors were induced in CBA mice (n = 15) by intramuscular (i.m.) injection of tumor cells (10^6^ cells/mouse) in 0.1 mL of PBS into the right thighs (n = 15) (Figure 1).

### 2.5. Neutrophil Phenotype Experiment

Before the first day of the experiment, mice were weighed and randomized according to their weight in several groups (control or treatment). Throughout the study, investigators responsible for data analysis were blinded to the group assignments of the mice.

On day 15 after LLC implantation and on day 21 after B16 and RLS_40_ implantation, mice were subdivided into subgroups according to tumor size. The mice were euthanized under isoflurane anesthesia and the spleens were collected for neutrophil isolation. Splenic neutrophils were isolated from the spleen of mice with LLC, RLS_40_, and B16, with primary tumor nodes reaching a volume of more than 1 cm^3^ at the end of the experiment, which corresponds to stable tumor progression.

On day 4 after RLS_40_ and B16 implantation, the mice were assigned to one of four groups (n = 20): (1) and (3)—tumor controls, mice with RLS_40_ (1) and B16 (3) received i.m. saline buffer (0.1 mL), respectively (groups RLS_40_ and B16); (2) and (4)—mice with RLS_40_ (2) and B16 (4) received i.m. DNase I (Thermo Fisher Scientific, Waltham, MA, USA) at a dose of 100 U/mice (0.1 mL, dissolved in saline buffer), respectively (groups RLS_40_/D and B16/D). Saline buffer or DNase I was administered on days 4–8, 11–15, and 19–21 (total number of administrations = 14). The dose and dosing frequency were determined based on a previous study [57].

On day 21, blood samples (up to 0.5 mL) were collected from the retro-orbital sinus 1 h after the last injection of saline buffer and DNase I. As controls, blood samples were collected from 15 healthy mice of the same age as those used in the experiment.

Mice with RLS_40_ were subdivided into subgroups according to tumor size. RLS_40_^High^ and RLS_40_^High^/D: tumor node more than 1 cm^3^, RLS_40_^Med^ and RLS_40_^Med^/D: tumor node more than 0.1 cm^3^ and less than 1 cm^3^, and RLS_40_^Low^ and RLS_40_^Low^/D: tumor node less than 0.1 cm^3^. In the subgroups RLS_40_^High^ and RLS_40_^Med^, the tumors were palpable, while in the RLS_40_^Low^ group, no palpable tumor nodes were observed.

A similar approach was used for mice with B16 from the DNase I experiment: the mice were divided into B16^High^ and B16^High^/D, as well as B16^Low^ and B16^Low^/D subgroups, in which the tumor nodes were more than 1 cm^3^ and less than 0.1 cm^3^, respectively.

The number of mice in each subgroup varied from 5 to 13, reflecting inherent variability in tumor growth that could not be experimentally controlled. To maintain consistency, 10 mice from each subgroup of different experiments (three replicates) were analyzed in the final dataset for all tests.

Then, the mice were euthanized under isoflurane anesthesia, spleens were collected from all subgroups of mice, and livers were collected from groups of mice with RLS_40_. Livers were fixed in 4% neutral-buffered formaldehyde (BioVitrum, St. Petersburg, Russia) for subsequent histological analysis. Spleens were used for neutrophil isolation. As a healthy control, bone marrow and spleen were collected from 10 healthy mice of the same age as those used in the experiment, as described previously [17].

### 2.6. Neutrophil Isolation

Bone marrow (BM) neutrophils (healthy controls) and splenic neutrophils (healthy or experimental) were isolated using the positive selection method on Dynabeads as described in [17]. Briefly, the BM was flushed using an insulin syringe contained RPMI/FBS/EDTA, while the spleens were homogenized mechanically in 1–3 mL of RPMI/FBS/EDTA to full dissociation. The cell suspension was filtered through a 70 μm cell strainer, and red blood cell lysis was performed using lysis buffer containing 0.15 M NH_4_Cl, 10 mM NaHCO_3_, and 0.1 mM EDTA (Sigma-Aldrich, Darmstadt, Germany). The cells were stained with rat IgG anti-mouse Ly6G (Elabscince, Houston, TX, USA, Cat#E-AB-F1108A) and then incubated with Dynabeads Sheep anti-Rat IgG (ThermoFisher Scientific, Waltham, MA, USA). Then, the neutrophils were isolated using a magnet (ThermoFisher Scientific, Waltham, MA, USA). The purity of the neutrophil samples assessed by flow cytometry analysis was >90%.

### 2.7. RNA Extraction

RNA was isolated from the cells using Rizol (diaGene, Moscow, Russia) according to the manufacturer’s instructions. The purity and integrity of the isolated RNA were assessed using NanoDrop^®^ oneC (Thermo Fisher Scientific, Waltham, MA, USA) and by visual inspection after gel electrophoresis.

### 2.8. Primer Design

The design of the primers and probes was performed using the RealTime PCR Tool supported by Integrated DNA Technologies (https://eu.idtdna.com/scitools/Applications/RealTimePCR/, accessed on 20 January 2024). Primers and probes were tested for self- and heterodimerization using the OligoAnalyzer™ Tool (https://eu.idtdna.com/pages/tools/oligoanalyzer, accessed on 20 January 2024). Potential secondary structures of the amplicons were analyzed using the MFOLD tool [58] (http://www.unafold.org/mfold/applications/dna-folding-form.php, accessed on 20 January 2024). Primers and probes were synthesized in the Laboratory of Biomedical Chemistry of ICBFM SB RAS (Novosibirsk, Russia). The primers used in this study are listed in Table S1.

### 2.9. cDNA Preparation and RT-qPCR

In the neutrophil phenotype experiment, using RT-qPCR mRNA levels of the genes *Fas*, *Icam1*, *Mmp9*, *Pd-l1*, *Sirt1*, *Tnfα,* and *Vegfa,* were determined in all groups (B16, LLC, and RLS_40_), while *Ccl17 and Il10* mRNA levels were determined for LLC and RLS_40_.

In the DNase I treatment experiment, the mRNA levels of the aforementioned genes for the RLS_40_ and B16 groups, respectively, were analyzed. Additionally, *Hgf*, *Ern1*, and *Stat3* were assessed in the B16 group, while these three genes plus *Vegfr1* were analyzed in the RLS_40_ group. Neutrophils isolated from the bone marrow and spleen of healthy mice of the corresponding mouse strains were used as controls.

Genes for RT-qPCR analysis were selected based on their assignment to one of the following marker groups: inflammation-related markers (anti-inflammatory pro-tumor markers *Ccl17*, *Il10,* and pro-inflammatory anti-tumor *Tnfa*), apoptosis-related markers (*Fas* and *Pd-l1*), pro-angiogenic and regenerative markers (*Hgf*, *Vegfa*, *Mmp9,* and *Vegfr1*), and participants of signaling pathways (*Ern1* and *Stat3*). We also studied the expression of neutrophil adhesion, migration marker *Icam1,* and NETosis suppresser *Sirt1*.

cDNA was prepared using reverse transcriptase M-MuLV–RH (Biolabmix, Novosibirsk, Russia). The reaction was carried out in 40 μL, which contained a total of 2 μg of isolated RNA, 200 U reverse transcriptase in RT buffer (Biolabmix, Novosibirsk, Russia), and a mixture of 1 μM (final concentration) dT18 and 1 μM random hexamer primers (Laboratory of Biomedical Chemistry of ICBFM SB RAS Novosibirsk, Russia). The reaction was carried out at 42 °C for 1 h and terminated by heating at 70 °C for 10 min. Reverse-transcribed RNA samples were diluted 10-fold and stored in aliquots at −80 °C.

The reaction mixture for qPCR (12.5 μL) contained 12.5 ng of cDNA, BioMaster HS-qPCR (BiolabMix), and 0.4 μM of each of the forward- and reverse-specific primers and 0.25 μM of the probe (Table S1). The RT-qPCR profile used was 95 °C for 6 min followed by 45 cycles of 95 °C for 15 s, 56 °C for 20 s, and 70 °C for 60 s. *Tbp* and *Hprt1* were used as reference genes [59]. The data were analyzed using the 2^−ΔΔCt^ method [60].

### 2.10. Flowcytometry Analysis

To investigate the phenotype of mouse neutrophils from the bone marrow and spleen of healthy mice and mice with tumors, the protein markers CD40, FAS, ICAM1, INFγR1, and PD-L1 were measured using flow cytometry after cell staining with respective monoclonal antibodies (Table S2). Between 2 × 10^5^ and 5 × 10^5^ neutrophils were used for each flow cytometry panel. The cells were pelleted (300 g for 10 min) and resuspended in PBS supplemented with 10% rat serum kindly provided by Dr. A.A. Seryapina (Institute of Cytology and Genetics SB RAS) for FC blocking and anti-Rat anti-IgG block on magnetic beads. After 5 min blocking, the antibodies were added (Table S2, Figure S1) and the samples were incubated for 20 min at room temperature in the dark. After staining, the cells were washed twice in PBS and analyzed using a NovoCyte 3000 flow cytometer (ACEA Biosciences, San Diego, CA, USA). Data were processed using NovoExpress software v. 1.1.0 (ACEA Biosciences, San Diego, CA, USA).

To determine the percentage of neutrophils in the spleen, 5 × 10^5^ splenocytes were stained for CD45, CD11b, and Ly6G (Table S2).

### 2.11. NETosis Induction

We studied the ability of splenic neutrophils isolated from RLS_40_^Low^ and RLS_40_^High^ subgroups (both control and experimental) to release NETs spontaneously or under activation with chemical activator A23187. Splenic neutrophils from healthy CBA mice were used as controls. For spontaneous NETosis, 10^5^ neutrophils were resuspended in 500 µL RPMI 1640 supplemented with 5 mM HEPES (Sigma-Aldrich, Darmstadt, Germany), 1 mM sodium pyruvate (ThermoFisher Scientific, Waltham, MA, USA), and 1% antibiotic-antimycotic solution (RPMI/HEPES/SP) [61,62]. For NETosis activation by calcium ionophore, 10^5^ neutrophils were resuspended in 500 µL of RPMI/HEPES/SP supplemented with calcium ionophore A23187 (Abcam, Cambridge, UK) at a final concentration of 5 µM.

### 2.12. Confocal Microscopy

For NETosis analysis, the cells were placed on circular cover glasses (13 mm diameter) pre-treated with poly-L-lysine solution (Sigma-Aldrich, Darmstadt, Germany) in the wells of a 24-well plate and incubated for 3 h at 37 °C under standard conditions. Then, 100 μL of 24% formaldehyde (Sigma-Aldrich, Darmstadt, Germany) was added to the wells (final concentration 4%) and incubated for 30 min at room temperature for cell fixation. After fixation, the coverslips were washed twice with PBS, treated with DIOC6 solution (0.6 μg/mL) (Cat#ab189808, Abcam, Cambridge, UK), and incubated at 37 °C in the dark for 20 min. Then, the coverslips were washed two times with PBS, treated with DAPI (1 μg/mL) (Cat# 62248, ThermoFisher Scientific, Waltham, MA, USA), and incubated at 37 °C in the dark for 20 min.

After incubation, the coverslips were washed with PBS, removed from the well, and flipped on a 10 μL of Fluoromount-G^TM^ mounting medium (00-4958-02 ThermoFisher Scientific, Waltham, MA, USA) placed on a glass slide. The slides were placed in the dark at room temperature in the horizontal position for one night and then analyzed by confocal fluorescence microscopy LSM710 (Zeiss, Munich, Germany) using a plan-apochromat 63×/1.40 Oil DIC M27 objective. The obtained images were analyzed using ZEN software 2012 (Zeiss, Munich, Germany) and ImageJ software version 1.54d (Wayne Rasband and contributors, NIH, Madison, WI, USA).

For NET quantification, the images were obtained using an EC Plan-Neofluar 20×/0.50 M27 objective, and cells in at least five non-overlapping fields were counted for each condition. Extended DNA filaments or decondensed cloud-like extracellular DNA were identified as NETs. The results are expressed as the percentage of neutrophils forming NETs. The percentage was calculated as the proportion of neutrophils producing NETs relative to the total number of neutrophils observed in the sample.

### 2.13. Neutrophil Effect on Tumor Cell Viability

Neutrophils isolated from RLS_40_-bearing mice treated with saline buffer or DNase I were used. Neutrophils and RLS_40_ cells in 0.5 mL FBS-free IMDM supplemented with antibiotic–antimycotic solution were placed in a 24-well plate in a 1:1 ratio (250,000 cells per well) and incubated for 24 h under standard conditions. Then, neutrophils were removed from the cell suspension using a magnet, and the level of RLS_40_ cell apoptosis was assessed by flow cytometry using Annexin V-FITC/PI staining (Apoptosis Detection Kit, Vazyme, Nanjing, China) as described in [63].

### 2.14. Peripheral Blood Analysis

Hematological parameters, such as total and differential leukocyte counts, were assessed in blood samples using a hematology analyzer (MicroCC20Vet; High Technology Inc., North Attleborough, MA, USA). Blood serum was prepared from fresh whole blood as described in [64].

Cell-free DNA (cfDNA) was isolated using a kit for the extraction of genomic DNA from cells, tissues, and blood (Biolabmix, Novosibirsk, Russia) according to the manufacturer’s recommendations. The concentration of cfDNA was quantified using a Qubit fluorometer (Invitrogen, Carlsbad, CA, USA) with a Quant-iT dsDNA HS Assay Kit (Thermo Fisher Scientific, Waltham, MA, USA), according to the manufacturer’s guidelines. The quality of cfDNA was assessed using a NanoDrop™ ND-1000 spectrophotometer (ThermoFisher Scientific, Waltham, MA, USA).

### 2.15. Determination of Nuclear and Mitochondrial DNA Quantity in cfDNA Samples by qPCR

Reaction mixture (20 μL) contained 0.1 ng of cfDNA, SYBR-Green-containing Bio Master CorHS-qPCR (BiolabMix, Novosibirsk, Russia), and 0.6 μM of each of the forward- and reverse-specific primers for the nuclear (*Actb*) and mitochondrial (*Nd3*) genes. The primer sequences are listed in Table S1. PCR was performed using a CFX96 Touch Real-Time PCR Detection System (Bio-Rad Laboratories Inc., Hercules, CA, USA). The reaction conditions were as follows: 95 °C, 6 min; 95 °C, 15 s; 60 °C, 20 s; 70 °C, 60 s; 40 cycles. Mitochondrial/nuclear DNA ratio was calculated as the ratio of the mitochondrial *Nd3* gene to nuclear *Actb* gene.

### 2.16. Histology and Immunohistochemistry

For the histological study, the tumor, liver, and thymus specimens were fixed in 10% neutral-buffered formalin (BioVitrum, Moscow, Russia), dehydrated in ascending ethanols and xylols, and embedded in HISTOMIX paraffin (BioVitrum, Russia). Paraffin sections (up to 5 µm) were sliced on a Microm HM 355S microtome (Thermo Fisher Scientific, Waltham, MA, USA) and stained with hematoxylin and eosin.

For the immunohistochemical study, the thymus sections were deparaffinized and rehydrated. Antigen retrieval was performed after exposure in a microwave oven at 700 W. The samples were incubated with anti-CD163 primary antibodies (ab182422, Abcam, Boston, MA, USA) according to the manufacturer’s protocol. Then, the sections were incubated with secondary horseradish peroxidase (HPR)-conjugated antibodies, exposed to the 3,30-diaminobenzidine (DAB) substrate (Rabbit Specific HRP/DAB (ABC) Detection IHC Kit, ab64261, Abcam, Boston, MA, USA), and stained with Mayer’s hematoxylin.

### 2.17. Morphometric Analysis

The morphometric analysis of liver sections included an assessment of the relative area of liver metastases indicating the percentages of the internal metastases areas in the liver relative to the total area of liver sections using Adobe Photoshop. Inhibition of metastases development was assessed using the metastasis inhibition index (MII), calculated as MII = ((mean metastasis area_control_ − mean metastasis area_experiment_)/mean metastasis area_control_) × 100%. MII in the tumor-bearing mice without treatment (control) was taken as 0%, and MII, reflecting the absence of metastases, was taken as 100%.

Morphometric analysis of tumor and thymus sections was performed by point counting using a morphometric grid with 100 testing points in a testing area equal to 3.2 × 10^6^ μm^2^. Morphometric analysis of tumor tissue included the assessment of the numerical density (Nv) of macrophages in the square unit. Morphometric analysis of thymus sections included the evaluation of the volume densities (Vv, %) of the cortex and medulla with subsequent calculation of thymus-cortex-to-medulla ratio as well as numerical density (Nv) of CD163^+^ macrophages.

The volume density (Vv, %) of the histological structure studied indicates that the volume fraction of tissue occupied by this compartment is determined from the testing points lying over this structure and is calculated using the following formula, Vv = (P_structure_/P_test_) × 100%, where P_structure_ denotes the number of points over the structure and P_test_ denotes the total number of test points, 100 in this case. The numerical density (Nv) of the histological structure studied indicates the number of particles in the unit of tissue volume and is evaluated as the number of particles in the square unit, 3.2 × 10^6^ μm^2^ in this case. From ten to fifteen random fields were examined from each liver, tumor, or thymus specimen.

All histological images were examined and scanned using an Axiostar Plus microscope equipped with an AxioCam MRc5 digital camera (Zeiss, Oberkochen, Germany) at magnifications ×100, ×200, and ×400.

### 2.18. Data Analysis

All analyses were performed with three technical replicates. Data on primary tumor growth inhibition were statistically processed using Student’s *t*-test (two tailed, unpaired). MII, metastasis number, and qPCR data were statistically processed using one-way ANOVA. Post hoc testing was performed using the post hoc Tukey test. Flow cytometry and RT-qPCR data were analyzed using the non-parametric Kruskal–Wallis test with Dunn’s multiple comparisons test and Bonferroni correction. *p* < 0.05 was considered statistically significant. The statistical package STATISTICA version 10.0 or GraphPad Prism version 8.0.2 were used for analysis.

## 3. Results

### 3.1. The Effect of Tumor Type on Neutrophil Phenotype

**Figure 2:**
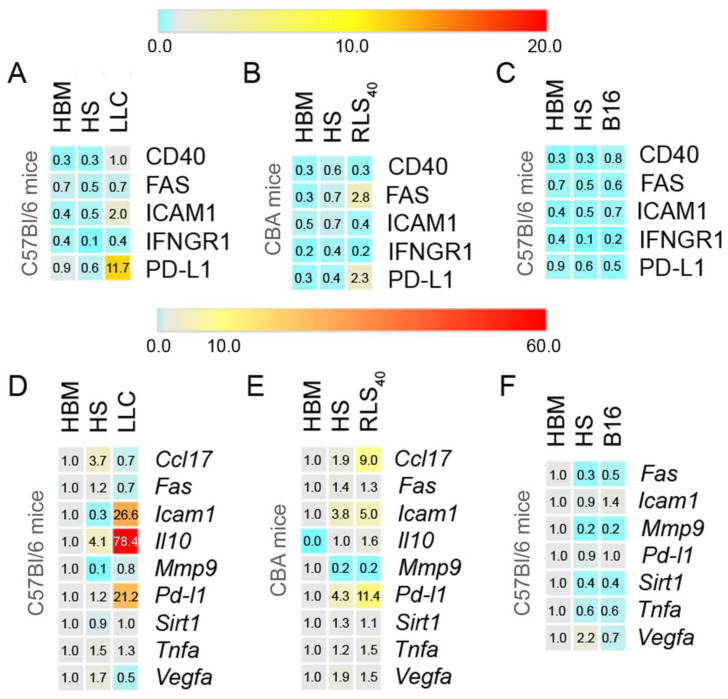
Heat maps showing the phenotype of neutrophils from the bone marrow and the spleen of healthy mice and spleen of mice with LLC (**A**,**D**), RLS_40_ (**B**,**E**), and B16 (**C**,**F**). (**A**–**C**) Flow cytometry data showing the percentage of cells expressing CD40, FAS, ICAM1, IFNGR1, and PD-L1 in the neutrophil gate (Ly6G^+^ events). (**D**–**F**) RT-qPCR data showing the levels of *Ccl17, Fas, Icam1, Il10, Mmp9, Pd-l1, Sirt1, Tnfa,* and *Vegfa* mRNA in neutrophils (2^−ΔΔCt^ analysis). HBM and HS: bone marrow and splenic neutrophils of healthy mice of respective mice strain, respectively.

The percentage of neutrophils of healthy mice expressing the studied protein markers varied slightly depending on the neutrophil origin, bone marrow or spleen, which confirms the need to use both as controls. It should be noted that the spleen neutrophils of healthy mice differed in the level of expression of protein markers and mRNA of selected genes depending on the mice strain (CBA/RLS_40_ and C57Bl/LLC and B16) and age of mice (compare HS data in Figure 2D–F). Another interesting observation was the slightly reduced mRNA level of some genes in splenic neutrophils relative to BM in younger mice, in particular (*Fas, Sirt1, Tnfa*, Figure 2F); additionally, we observed that all splenic neutrophil samples from healthy mice had reduced levels of *Mmp9* mRNA compared to those in BM neutrophils.

Tumor development is accompanied by the acquisition of splenic neutrophil phenotypes quite different from those observed in HS. The most noticeable enhancement of splenic neutrophil population expressing PD-L1 was observed upon LLC development (from 0.6 ± 0.02% to 11.7 ± 0.8%) (Figure 2A). This result was consistent with the RT-qPCR data, where the level of *Pd-l1* mRNA was 18-fold-increased in comparison with HS (Figure 2D). Moreover, in the LLC group, there was a 19-fold increase in *Il10* and a 26-fold increase in *Icam1* mRNA levels (Figure 2D) compared with HS neutrophils. This is consistent with the flow cytometry results showing that the number of ICAM^+^ neutrophil population increased approximately four-fold compared with HS samples (Figure 2A).

During the development of RLS_40_, a similar trend was observed: the increase in *Pd-l1* mRNA level from 4.3 ± 0.5 to 11.4 ± 1.9 and the increase in PD-L1^+^ cell population from 0.4 ± 0.1 to 2.3 ± 0.2% (compared to HS neutrophils, Figure 2B,E). RLS_40_ progression resulted in a 1.3-fold increase in *Icam1* and a 4.7-fold increase in *Ccl17* mRNA expression levels (compared to HS neutrophils, Figure 2E). Flow cytometric analysis showed an insignificant elevation (0.2–0.4%) of CD40^+^, ICAM1^+^, and IFNGR1^+^ populations (Figure 2B).

B16 development did not lead to significant changes in the mRNA and protein marker levels. Flow cytometry analysis did not reveal any changes in the percentage of neutrophils expressing the studied markers (Figure 2C). RT-qPCR data also showed no significant changes in the expression level of the studied genes, excluding *Icam1*, which increased by 1.6-fold (Figure 2F).

### 3.2. The Effect of DNase I on Tumor Progression, Immune Response and Neutrophil Phenotype of Mice with RLS_40_ and B16

#### 3.2.1. The Effect of DNase I on Tumor Growth and Metastases Development in Mice with RLS_40_ and B16

**Figure 3:**
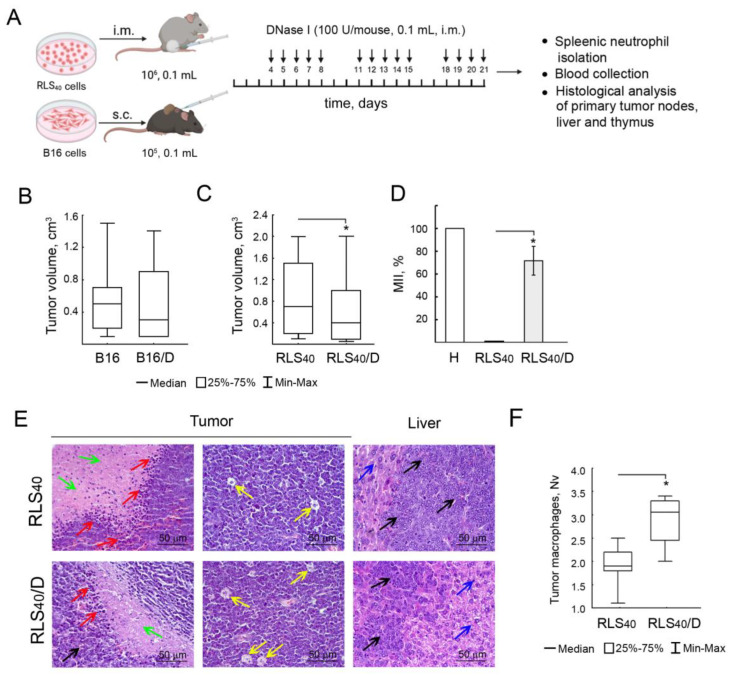
Effect of DNase I treatment on RLS_40_ and B16 tumor growth and metastases development. (**A**) Experimental scheme. RLS_40_ cells were implanted intramuscularly (i.m., 10^6^ cells, 0.1 mL) into CBA mice, and B16 cells were implanted subcutaneously (s.c., 10^5^ cells, 0.1 mL) into C57Bl mice. Starting from day 4, animals received i.m. saline buffer or DNase I (100 U/mouse) daily, except for the weekend (5 + 2). (**B**,**C**) Tumor volumes in mice with B16 and RLS_40_, respectively. The data are presented as medians. Data were statistically analyzed using one-way ANOVA with a post hoc Tukey test. Statistical significance * is *p* < 0.05. (**D**) Metastases in the liver of mice with RLS_40_. MII (metastases inhibition index) = ([mean metastasis area_control_−mean metastasis area_experiment_]/mean metastasis area_control_) × 100%. Data are presented as mean ± SEM. (**E**) Representative histological images of primary tumor nodes and liver metastases of mice with RLS_40_. Hematoxylin and eosin staining. Original magnification ×400. Black arrows indicate tumor tissue and liver metastases, green arrows—necrotic decay, red arrows—cell infiltration, yellow arrows—tumor macrophages, and blue arrows—liver tissue. (**F**) Numerical density (Nv) of macrophages in the tumor tissue of primary tumor nodes.

DNase I administration exhibited no effect on the size of the B16 tumor node but caused a two-fold decrease in the RLS_40_ tumor volume (Figure 3B,C). Moreover, DNase I treatment showed a reliable antimetastatic effect: the metastasis inhibition index (MII) was 70% in the RLS_40_/D subgroup that received DNase I compared with the control (MII 0%) (Figure 3D).

Histological analysis showed that RLS_40_ tumor nodes and liver metastases were represented by polymorphic lymphoid cells with necrotic decay in the central areas of tumor nodes and reactive cell infiltration represented predominately by lymphocytes and macrophages on the border with unchanged tumor tissue (Figure 3E). The morphometric study of tumor nodes of the control group demonstrated the presence of macrophages outside the areas of tumor tissue destruction amounting to 1.9 ± 0.2 per test area. DNase I administration led to a 1.5-fold increase in this parameter compared with the control (Figure 3E,F).

#### 3.2.2. Analysis of Concentration of Blood Serum cfDNA and Mitochondrial/Nuclear DNA Ratio

Analysis of blood serum cfDNA showed a strong correlation between tumor volume and cfDNA concentration: a significant 7.4-fold increase in cfDNA concentration was detected in the RLS_40_^High^ subgroup, and a 4-fold increase in the RLS_40_^Med^ and RLS_40_^Low^ subgroups in comparison with healthy mice (Table 1). In the DNase I-treated groups, the concentration of cfDNA decreased, but, as expected, a more pronounced effect was observed in the RLS_40_^Med^/D and RLS_40_^Low^/D subgroups. It should be noticed that, even in these groups, cfDNA concentration was twice as high as that in healthy CBA mice (Table 1). In the B16 model, upon tumor development, the concentration of cfDNA increased three- and two-fold in B16^High^ and B16^Low^ mice, respectively, in comparison with the healthy control, and DNase I treatment had no effect on this parameter, which is consistent with the lack of DNase I-suppressing activity on B16 tumor growth (Figure 3B).

An interesting result was obtained for the dynamics of the ratio of mitochondrial (mtDNA) and nuclear DNA (nucDNA). In healthy mice, the pool of cfDNA contains mitochondrial DNA, but its quantity is relatively low. Tumor development is accompanied by a 10-fold increase in the mtDNA/nucDNA ratio (Table 1). Interestingly, both spontaneous (RLS_40_^Low^) and DNase-mediated tumor reduction (RLS_40_^Low^/D) are accompanied by an increase in the mtDNA/nucDNA ratio, with the highest ratio observed in the RLS_40_^Low^/D subgroup. In the case of the B16 model, the same increase in the mtDNA/nucDNA ratio was found upon tumor development, but only an insignificant fluctuation in this ratio was found after DNase I treatment (Table 1).

#### 3.2.3. Alterations in Immunocompetent Organs

**Figure 4:**
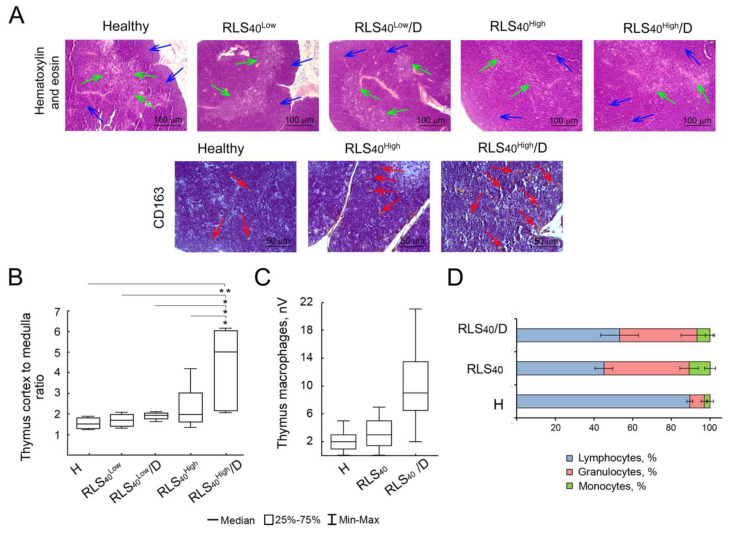
Effect of DNase I treatment on immune cells in the thymus and peripheral blood of mice with RLS_40_. (**A**) Representative histological images of the thymus of healthy control and experimental mice. Hematoxylin and eosin staining (**upper** panel) and immunohistochemical staining with anti-CD163 primary antibody (**bottom** panel). Blue arrows indicate thymus cortex, green arrows indicate thymus medulla, and red arrows indicate CD163^+^ thymus macrophages. (**B**) Thymus-cortex-to-medulla ratio in healthy, RLS_40_, and RLS_40_/D subgroups. (**C**) Numerical density of CD163^+^ thymus macrophages. (**D**) Proportion (%) of immune cells in the peripheral blood of healthy, control, and experimental mice. * *p* ≤ 0.05; ** *p* ≤ 0.01.

In healthy animals, the thymus cortex was amounted 59.8 ± 2.4%, and the thymus-cortex-to-medulla ratio in this group was 1.5 ± 0.2 (Figure 4A,C). The tumor growth in control subgroups RLS_40_^Low^ and RLS_40_^High^ led to an increase in thymus cortex of up to 67.4 ± 2.6% and 62.0 ± 1.5%, respectively; thus, the cortex-to-medulla ratio for these groups increased up to 2.3 and 2.0, respectively, reflecting the immune system activation by the tumor progression (Figure 4A,B). In the RLS_40_^High^/D subgroup, an increase in thymus cortex area was detected, whereas, in the RLS_40_^Low^/D, only a slight insignificant increase was found; the cortex-to-medulla ratio in both subgroups was 4.4 ± 0.8 and 2.1 ± 1.9, respectively. Morphometric analysis of thymus CD163^+^ macrophages revealed a significant 5.3- and 3.3-fold increase in this parameter after DNase I administration compared to healthy and tumor-bearing mice without treatment (10.7 ± 1.7 vs. 2.0 ± 0.5 and 3.2 ± 0.5, respectively, Figure 4C), indicating stimulation of the immune system not only by the tumor process, but also by treatment with DNase I (Figure 4A,C).

Analysis of the peripheral blood of control and experimental mice showed a 5.9- and 5.3-fold increase in the proportion of granulocytes, as well as a 3.6- and 2.7-fold increase in the proportion of monocytes compared to healthy animals, reflecting a response of the immune system to tumor development (Figure 4D).

#### 3.2.4. The Effect of DNase I on Neutrophil Phenotype of Mice with RLS_40_ and B16

**Figure 5:**
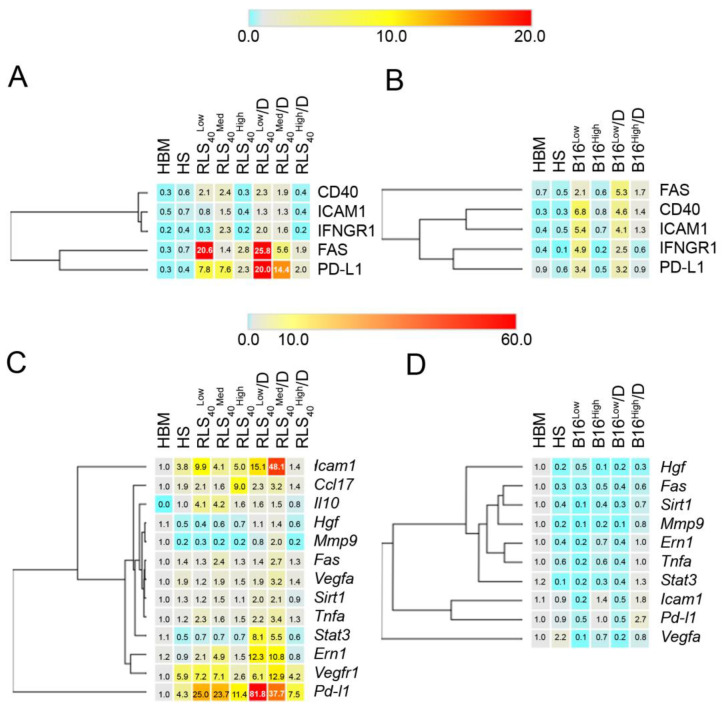
Heat map showing the phenotypes of bone marrow and splenic neutrophils isolated from healthy mice and splenic neutrophils isolated from mice with RLS_40_ (**A**,**C**) or B16 (**B**,**D**) that received saline buffer (RLS_40_^Low^, RLS_40_^Med^, and RLS_40_^High^) and DNase I (RLS_40_^Low^/D, RLS_40_^Med^/D, and RLS_40_^High^/D). (**A**,**B**) Flow cytometry results showing % of cells expressing CD40, Fas, ICAM1, IFNGR1, and PD-L1 in the neutrophil gate (Ly6G^+^ events). (**C**,**D**) PCR results showing different mRNA levels in neutrophils (2^−ΔΔCt^ analysis). HBM and HS: bone marrow and splenic neutrophils from healthy mice, respectively.

In the B16 model, DNase treatment had no effect on the neutrophil phenotype: no changes in the protein markers or in the mRNA levels of the studies genes were observed. At the same time, in the RLS_40_ model, DNase treatment resulted in a decrease in the mRNA levels of pro-tumor phenotype markers (*Ccl17, Il10*) and an increase in the mRNA levels of anti-tumor phenotype markers (*Icam1, Tnfa, Vegfr1*), as well as cell mobilization genes (*Ern1, Stat3*). Quite unexpectedly, a significant increase in *Pd-l1* mRNA level was observed, and, according to the flow cytometry results, DNase I treatment resulted in an increase in the number of neutrophils with potential anti-tumor phenotype (FAS^+^ and PD-L1^+^) (Figure 5).

In the RLS_40_ group, the most interesting observation was the alterations in the expression of PD-L1 depending on the tumor volume observed at both the protein and mRNA levels (Figure 5A,C). In the control subgroups, an increase in *Pd-l1* mRNA expression negatively correlated with tumor volume: *Pd-l1* mRNA levels in the RLS_40_^High^ subgroup were the lowest (11.4 ± 1.9), and the highest increase in *Pd-l1* mRNA expression was detected in the RLS_40_^Med^ and RLS_40_^Low^ subgroups (23.7 ± 1.2 and 25.0 ± 4.3, respectively). DNase I treatment enhanced *Pd-l1* mRNA level, amounting to 81.8 ± 12.5 in the RLS_40_^Low^/D subgroup and 37.7 ± 1.3 in the RLS_40_^Med^ subgroup, while, in the RLS_40_^High^/D subgroup, no effect was observed (Figure 5C). This increase in gene expression was consistent with neutrophil population expressing the PD-L1 protein (PD-L1^+^), which increased in the same manner. Treatment with DNase I caused a two–three-fold increase in PD-L1^+^ population in the RLS_40_^Low^/D and RLS_40_^Med^/D subgroups in comparison to the corresponding control group (RLS_40_^Low^ and RLS_40_^Med^), and it did not change its percentage in the RLS_40_^High^/D subgroup (Figure 5A).

A similar tendency was observed for the neutrophil population expressing FAS protein. The higher the tumor volume in the RLS_40_ control subgroups, the lower the percentage of FAS^+^ neutrophil population: 20.6 ± 1.9% in the RLS_40_^Low^ subgroup and 1.4 ± 0.1 and 2.8 ± 0.3 in RLS_40_^Med^ and RLS_40_^High^ subgroups, respectively (Figure 5A). DNase I administration affecting tumor size led to an increase in FAS^+^ population to 25.8 ± 8.9% in the RLS_40_^Low^/D and to 5.6 ± 1.5% in the RLS_40_^Med^/D subgroup. However, *Fas* mRNA levels were unchanged in all subgroups, both control and experimental, in comparison to HS neutrophils, but some insignificant fluctuations of *Fas* mRNA expression were observed (Figure 5C), which could be explained by the very short lifetime of this mRNA.

The levels of other protein markers showed no alterations upon tumor development and treatment according to flowcytometry data, while RT-qPCR analysis showed that the expression of some other genes was affected by tumor growth and DNase I treatment.

According to RT-qPCR data, the most pronounced changes in expression were observed for *Icam1, Ern1*, *Vegfa*, *Ccl17*, *Il10,* and *Stat3* genes. However, in most cases, these alterations, in particular, mRNA levels, did not correlate with tumor volume and/or DNase I treatment (Figure 5C). In the RLS_40_ subgroups, an increased level of *Icam1* mRNA (2.6-fold) was found in the RLS_40_^Low^ subgroup in comparison with HS (9.9 ± 2.1 and 3.8 ± 2.5%, respectively), whereas no significant changes in *Icam1* mRNA level were observed in the RLS_40_^Med^ and RLS_40_^High^ subgroups (Figure 5C). In the RLS_40_/D subgroups, mRNA *Icam1* was increased in the RLS_40_^Low^/D and RLS_40_^Med^/D subgroups (15.1 ± 2.8 and 48.1 ± 0.6, respectively), while no changes in the RLS_40_^High^/D subgroup were detected (Figure 5C).

An increased expression of the anti-inflammatory pro-tumor gene *Ccl17* was observed only in the RLS_40_^High^ subgroup, while, in DNase I-treated subgroups, *Ccl17* mRNA expression was at the level observed in the healthy control (Figure 5C). Similarly, the expression of *Il10*, which encodes another anti-inflammatory pro-tumor marker, was slightly upregulated (up to four-fold compared to HS) in the RLS_40_^Low^ and RLS_40_^Med^ subgroups and at the level of healthy control (HS) in the RLS_40_^High^ and RLS_40_/D subgroups (Figure 5C). The expression levels of the pro-inflammatory anti-tumor marker *Tnfa* were a little increased by up to two–three-fold in the RLS_40_^Low^, RLS_40_^Low^/D, and RLS_40_^Med^/D subgroups that were not dependent on DNase I treatment (Figure 5C).

By analyzing the regenerative and angiogenic markers (*Mmp9*, *Vegfa*, *Vegfr1*), we found no significant changes in *Mmp9* expression in all control and experimental subgroups. Similarly, no changes were found in *Vegfa* expression: insignificant fluctuations of *Vegfa* mRNA level were observed in all control and experimental subgroups, excluding the RLS_40_^Med^/D subgroup, in which an increase was detected in comparison to RLS_40_^Med^ (3.2 ± 0.1 vs. 1.9 ± 0.1) and HS (3.2 ± 0.1 vs. 1.9 ± 0.2) (Figure 5C). The level of *Vegfr1* mRNA, the product of which is the receptor of VEGF, varied between subgroups: we detected a slight fluctuation in *Vegfr1* mRNA levels in the RLS_40_^Low^ and RLS_40_^Low^/D subgroups (7.2 ± 2.1 vs. 6.1 ± 2.9, HS = 5.9 ± 0.1), its essential increase in RLS_40_^Med^/D in comparison with the RLS_40_^Med^ subgroup (12.9 ± 5.0 vs. 7.1 ± 0.5, HS = 5.9 ± 0.1), and its decrease in the RLS_40_^High^ and RLS_40_^High^/D subgroups in comparison with HS (2.6 ± 0.6 vs. 4.2 ± 0.8, HS = 5.9 ± 0.1).

Among cell mobilization markers, *Ern1* mRNA expression level was increased both in RLS_40_^Low^/D in comparison with the RLS_40_^Low^ subgroup (12.3 ± 6.8 vs. 2.1 ± 0.8, HS = 0.9 ± 0.2) and RLS_40_^Med^/D in comparison with the RLS_40_^Med^ subgroup (10.8 ± 5.9 vs. 4.9 ± 1.8, HS = 0.9 ± 0.2), and it slightly fluctuated in the RLS_40_^High^ and RLS_40_^High^/D subgroups (1.5 ± 0.2 vs. 0.8 ± 0.4, HS = 0.9 ± 0.2)*. Stat3* was only moderately overexpressed in the RLS_40_^Low^/D and RLS_40_^Med^/D subgroups (8.1 ± 4.4 and 5.5 ± 3.0, HS = 0.5 ± 0.1) (Figure 5C).

In the B16 model, minimal alterations (2–7%) of the neutrophil population expressing the studied markers (CD40, FAS, ICAM1, IFNɣR1, and PD-L1) in the B16^Low^ and B16^Low^/D subgroups, independent of DNase I treatment, were observed (Figure 5B). RT-qPCR data showed downregulation of most studied genes in all B16 subgroups, except for *Icam1* and *Pd-l1* (Figure 5D). We observed an increase in *Icam1* mRNA levels in the B16^High^ and B16^High^/D subgroups (1.4 ± 0.6 and 1.8 ± 0.5, HS = 1.1 ± 0.3) and an increase in *Pd-l1* mRNA expression level in B16^High^/D in comparison with the B16^High^ subgroup (2.7 ± 1.2 vs. 1.0 ± 0.03, HS = 1.0 ± 0.1).

#### 3.2.5. The Effect of DNase I on the Ability of Neutrophils to Generate NETs

**Figure 6:**
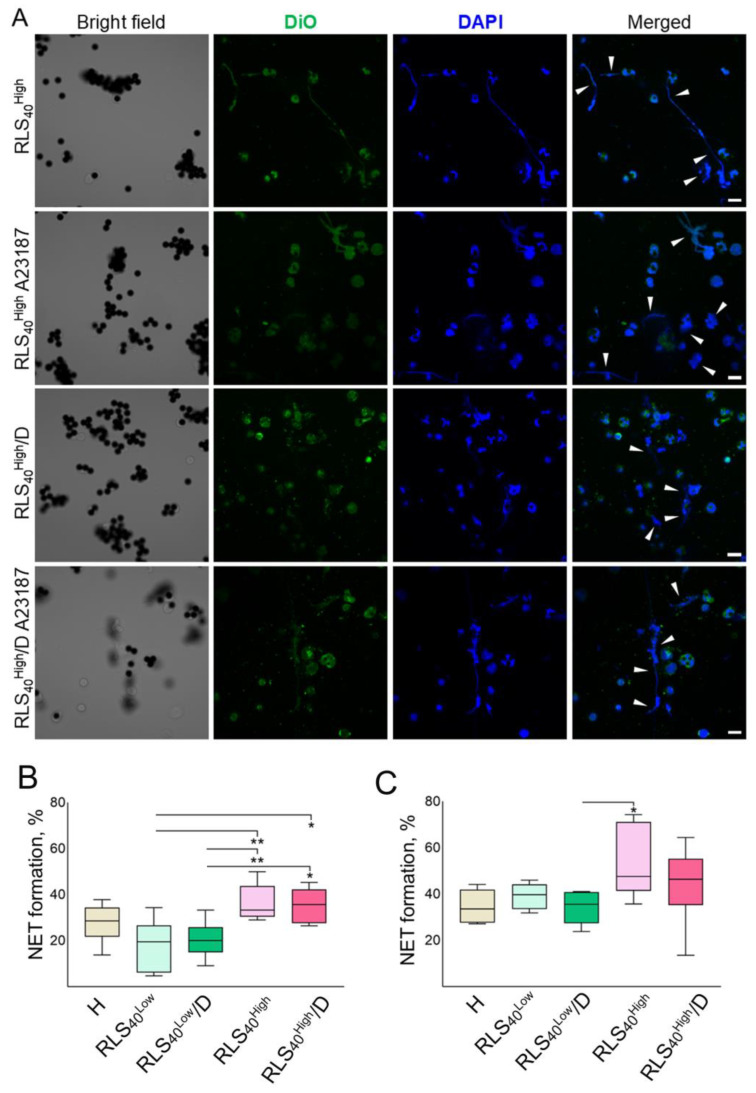
NETs generated by splenic neutrophils isolated from mice with RLS_40_. (**A**) Confocal microscopy images of NET formation by splenic neutrophils isolated from RLS_40_-bearing mice spontaneously or under A23187 stimulation. Magnification ×63. (**B**) Percentage of splenic neutrophils subjected to spontaneous NETosis. (**C**) Percentage of splenic neutrophils subjected to NETosis under A23187 activation. The quantitative data of NET formation were calculated as the percentage of neutrophils releasing filament-like or decondensed cloud-like NETs (white arrows). Data of confocal fluorescent microscopy. Scale bar 10 μM. Ordinary one-way ANOVA with Tukey’s multiple comparisons test was used. * *p* ≤ 0.05, ** *p* ≤ 0.01.

Representative confocal florescent microscopy images are displayed in Figure 6A. The presented data show that neutrophils isolated from the RLS_40_^Low^ and RLS_40_^High^ subgroups, whether naïve or stimulated by A23187, undergo NETosis, characterized by the formation of filament-like or cloud-like NETs (DAPI and Merged panels, white arrows, Figure 6A).

The level of spontaneous NETosis in the neutrophils of healthy mice was 20% (Figure 6B). Spontaneous NET formation was elevated in the RLS_40_^High^ and RLS_40_^High^/D subgroups compared to the RLS_40_^Low^ subgroup: spontaneous NET formation was 33% (range 29–50%) in the RLS_40_^High^ subgroup and 36% (range 26–45%) in the RLS_40_^High^/D subgroup (Figure 6B). Neutrophils from the RLS_40_^Low^ and RLS_40_^Low^/D subgroups released the lowest levels of NETs under spontaneous NETosis with a median of 20% for both groups (range 5–34% and 9–33%, respectively) (Figure 6B).

Under A23187 stimulation, all groups responded to the activation, and higher NET formation levels were observed in comparison with spontaneous NETosis (Figure 6C compared to B). The level of stimulated NETosis in neutrophils of healthy mice and the RLS_40_^Low^ and RLS_40_^Low^/D subgroups was approximately the same and amounted to 38% (Figure 6C). After stimulation, neutrophils from RLS_40_^High^ and RLS_40_^High^/D mice were characterized by higher levels of NET formation in comparison with other groups: NETosis levels were 48% (range 36–74%) and 46% (range 14–64%) in RLS_40_^High^ and RLS_40_^High^/D, respectively (Figure 6C).

## 4. Discussion

In this study, we analyzed the phenotype of splenic neutrophils isolated from tumor-bearing mice and investigated the effects of DNase I treatment on the phenotype and functionality of splenic neutrophils. The three mouse models used in this study included classical LLC, resistant lymphosarcoma RLS_40_, and subcutaneous melanoma B16-F10. LLC is of epithelial origin, is homologous to human squamous cell lung carcinoma, and metastasizes into the lungs [65]. RLS_40_ is of hematopoietic origin and is homologous to human diffuse large B-cell lymphoma [66,67]. B16 is of neural origin, represents degenerated melanocytes, and is homologous to human metastatic melanoma [68]. B16 is distinguished for its poor immunogenicity and low immune cell infiltration [69,70]. All three tumors are aggressive and form upon the implantation of rather big tumor nodes by 14-21 days. Unlike B16 and LLC, RLS_40_ was shown to be sensitive to the immune status of animals, leading to a spontaneous reduction in tumor volume [71]. In the cases of both large and reduced tumor nodes, RLS_40_ metastasizes to the liver [67].

*The impact of various tumor types on the phenotype of splenic neutrophils*. The markers of neutrophils studied in our work relate to key neutrophil functions during tumor progression. These markers identified for tumor-associated neutrophils included those associated with inflammation, such as anti-inflammatory pro-tumor markers (*Ccl17*, *Il10*) and pro-inflammatory anti-tumor markers (*Tnfα*, *Icam1*, CD40, and IFNɣR1); markers associated with apoptosis or NETosis (*Fas* and *Cd274* (PD-L1), *Sirt1*); pro-angiogenic and regenerative markers (*Vegfa*, *Vegfr1*, *Hgf,* and *Mmp9*); and participants of signaling pathways (*Ern1* and *Stat3*).

As was expected, we found a pro-tumor N2 neutrophil population in the spleen of mice with LLC. Splenic neutrophils from mice with LLC exhibited immunosuppressive features (high expression of the anti-inflammatory cytokines IL10 and PD-L1, which are mediators of immunosuppression) (Figure 2A,D). Il10, produced by TANs, contributes to immunosuppressive TME creation by inhibiting cytotoxic NK and CD8^+^ T cells [4,72]. PD-L1 could also contribute to tumor immunosuppression by inducing apoptosis in activated T cells [73]. Interestingly, expressions of other markers characteristic of human pro-tumor neutrophils, like MMP9, VEGFa, and CCL17, were not detected (Figure 2D). The anti-tumor N1 marker ICAM1 was detected at the mRNA level, but only a minimal ICAM1^+^ neutrophil population (1%) was observed. ICAM1 is a key player in neutrophil adhesion and is associated with neutrophil anti-tumor phenotype [74]. This confirms that pro-tumor neutrophils predominate in the late stages of tumor development, but anti-tumor and transitional neutrophils expressing both N1 and N2 markers can coexist within the spleen.

Similar results were obtained using the RLS_40_ model. In the spleens of RLS_40_-bearing mice (tumor node > 1 cm^3^), the expression of markers corresponding to immunosuppressive phenotype were detected: in addition to PD-L1, the expression of CCL17 but not IL10 was detected (Figure 2B,E). CCL17 contributes to tumor immunosuppression by recruiting Tregs to the TME [75]. Increased *Icam1* mRNA levels (an anti-tumor N1 phenotype marker) were also observed, although no corresponding neutrophil population (ICAM^+^) was detected by flow cytometry. Interestingly, PD-L1^+^ neutrophils were FAS^+^, despite no change in *Fas* mRNA levels being found by RT-qPCR (likely due to the transcript’s short lifespan) (Figure 2B,E). FAS is considered an anti-tumor marker of neutrophils that triggers tumor cell apoptosis by interacting with its ligand FASL on tumor cells [76,77,78]. Moreover, FAS on neutrophils participates in neutrophil adhesion and slow rolling in blood vessels via interaction with its ligand on endothelial cells [79].

An important observation is the insignificant alteration of the phenotype of neutrophils isolated from B16-bearing mice (tumor node > 1 cm^3^), where no significant increase in expression was found for any of the studied genes (Figure 2C,F). Thus, the B16 model did not show the common feature observed in the case of both RLS_40_ and LLC models with standard tumor size (tumor node ≥ 1 cm^3^), where splenic neutrophils could exhibit moderate or high expression of the adhesion marker *Icam1* (at the mRNA level), higher PD-L1 expression, and immunosuppressive markers (*Il10* mRNA for LLC and *Ccl17* mRNA for RLS_40_).

While, in LLC tumor model, tumor nodules larger than 1 cm^3^ are developed in all animals in the group, the RLS_40_ model is more variable and RLS_40_ tumor node size varies, ranging from small (less than 0.1 cm^3^, RLS_40_^Low^), corresponding to tumor growth controlled by the immune system, to large (more than 1 cm^3^, RLS_40_^High^), indicating tumor escape from immune surveillance, as well the transitional state RLS_40_^Med^ (tumor nodules between 0.1 and 1 cm^3^ in size), representing the transition from immune control to immune escape.

*Neutrophil phenotype alteration relative to tumor growth rate and treatment.* In the next step, we investigated whether spleen neutrophil profiles differ between mice bearing small and large tumors, to assess whether tumor growth intensity correlates with cfDNA levels and neutrophil phenotype, hypothesizing that mice with small tumors could exhibit a stronger immune response and a higher proportion of neutrophils with an anti-tumor phenotype.

To study whether treatment with a drug possessing anti-tumor and antimetastatic properties would affect the splenic neutrophil profile, and to enhance the differential phenotype of neutrophils depending on tumor growth rate and overall immune response, we used bovine pancreatic DNase I, which previously was shown to exhibit a strong antimetastatic effect and a relatively modest anti-tumor effect in a number of tumors. It was demonstrated that DNase I, when administered intramuscularly, produced an antimetastatic effect in the LLC model and in metastatic models of B16 and A1 hepatoma and a slight anti-tumor effect in the LLC model [42,43,44,46,47]. However, using the RLS_40_ model, both anti-tumor and antimetastatic effects of DNase I were demonstrated [47]. The effect of DNase I on tumor node size in the melanoma model has not been previously studied and represents an interesting research question, especially considering the fact that mice bearing B16 also exhibit clear differences in tumor node size linked to their immune system activity. Therefore, we studied the dependence of the neutrophil phenotype on the response of mice with RLS_40_ and B16 to treatment with DNase I.

No significant effects of DNase I treatment were found in the B16 model. Although the median tumor size decreased following DNase treatment, this reduction was not statistically significant (Figure 3B). Moreover, after DNase treatment, in splenic neutrophils, no significant changes in gene expression were detected for any of the studied genes. At the protein level, a slight increase in marker expression was observed for all of the studied markers in the B16^Low^ and B16^Low^/D subgroups; however, no significant differences were found between subgroups before and after treatment or compared to controls (Figure 5B,D).

Using a metastatic model of B16, we previously showed that DNase I, administered intramuscularly and intranasally, significantly reduces the number of lung metastases, accompanied by non-specific immunostimulation [42,43]. However, in a B16 model with a primary tumor node, we noticed neither a reduction in the tumor node size nor immunostimulation following DNase I treatment. Additionally, there was no significant decrease in cfDNA concentration (Table 1). A possible explanation for the lack of effect of DNase I in this model is impaired immune crosstalk during tumor development, particularly between T cells and neutrophils, which is crucial for effective anti-tumor immunity. Activated T cells engage neutrophils in anti-tumor immune responses [80,81,82]. Low tumor immunogenicity, the tumor’s ability to induce an adaptive immune response, grants tumor growth and metastasis spread without immune recognition [83]. Tumor immunogenicity depends on tumor-associated antigens (TAA) presented on MHC class I molecules, recognized by CD8+ cells that trigger apoptosis, cell cycle arrest, and the release of anti-tumor mediators like IFN-γ and TNF-α [84]. B16 melanoma is known for its low (or “no”) immunogenicity, which could be explained by the low levels of MHC I on B16 cells [70]. In addition, B16 tumor nodes are characterized by low immune cell infiltration, which could impair any adaptive immune responses, if any are present. [69,70]. The low immunogenicity and cell infiltration of B16 tumors could explain the observed results for splenic neutrophil phenotype and low response to DNase I treatment.

Unexpected promising results were obtained in the RLS_40_ model within the RLS_40_^Low^ and RLS_40_^Med^ subgroups (both control and treatment). An interesting finding was that the dynamic increase in PD-L1 expression negatively correlated with tumor size: a slight increase was observed in the RLS_40_^High^ group, a higher increase in the RLS_40_^Med^ group, and the highest increase in the RLS_40_^Low^ group. Notably, DNase I treatment further enhanced PD-L1 expression, primarily in the RLS_40_^Low^/D and, to a lesser extent, in the RLS_40_^Med^/D subgroups, whereas no effect was observed in the RLS_40_^High^/D subgroup (Figure 5A,C). The role of PD-L1 on neutrophils is not fully understood. As described previously, PD-L1 is considered a pro-tumor marker due to its ability to induce apoptosis in activated T cells [73]. However, anti-tumor neutrophils also express PD-L1, indicating its subtle role in neutrophil function in cancer [85]. Although PD-L1^+^ neutrophils are generally considered pro-tumor players in the TME, in the case of RLS_40_, PD-L1 could be a positive marker since RLS_40_ cells of lymphatic origin express PD-1 on their surface. Similar results were observed for FAS, which is regarded as an anti-tumor neutrophil marker. In the RLS_40_^Low^/D and RLS_40_^Med^/D subgroups, there was evidence of increased neutrophil adhesion ability, indicated by elevated *Icam1* expression accompanied by *Vegfr1* expression.

Thus, neutrophils with dynamically altered phenotypes appear in the spleen depending on immune system activity and treatment response. The general immunosuppressive phenotype found in the untreated control subgroup with large tumors (RLS_40_^High^), characterized by an increased expression of PD-L1 and CCL17, no IL10 expression, and a slight increase in the adhesion marker ICAM1, was changed in the control group with small tumors (RLS_40_^Low^, increased expression of PD-L1, CCL17 expression disappeared, and IL10 expression rose). Moreover, the expression of N1 phenotype markers increased, with ICAM1 and FAS showing a more pronounced rise, whereas CD40 increased to a lesser extent. Neutrophils directly interact with platelets through many surface proteins and can form heterogenic aggregates [86]. CD40, expressed on neutrophils, participates in this direct interaction by binding to CD40L on activated platelets or to soluble CD40L produced by platelets [87,88]. This interaction activates neutrophils and enhances their anti-tumor functions [88,89].

After DNase treatment, neutrophils are characterized by increased PD-L1 expression, but they lacked other immunosuppressive markers, such as CCL17 and IL10. This suggests that PD-L1 could function not as an immunosuppressive marker, but more as an anti-tumor factor specifically targeting PD-1-expressing lymphosarcoma cells. This interpretation is supported by clustering analysis (flowcytometry data), which groups PD-L1 with N1 markers. Along with PD-L1, the expression of the N1 marker ICAM1 is increased, along with an insignificant increase in STAT3 and ERN1 expression. ERN1 and STAT3 are important signaling molecules. In neutrophils, ERN1 is a key regulator of immune responses [90], while STAT3 signaling controls neutrophil mobilization [91]. However, some studies have shown that both molecules participate in pro-tumor neutrophil functions [92,93]. However, in this context, it is likely to reflect a general activation and mobilization of neutrophils.

Neutrophils were shown to mediate pro-angiogenic tumor-supporting functions. The most common markers of this phenotype are VEGFA, VEGFR1, HGF, and MMP9 [94,95,96]. These functions mostly arise from the regenerative roles of neutrophils, which are exploited by cancer cells to support tumor growth and metastasis [97]. The insignificant increase in *Vegfr1* expression in the RLS_40_^Low^ and RLS_40_^Med^ groups, as well as their DNase-treated counterparts (RLS_40_^Low^/D and RLS_40_^Med^/D), is noteworthy. We suggest that this upregulation may be important for enhancing the adhesive properties of potentially anti-tumor neutrophils rather than pro-angiogenic ones. At the same time, MMP9 expression is absent across all groups, and VEGFA changes insignificantly, indicating that neutrophils play a minimal role in extracellular matrix remodeling and angiogenesis during the development of RLS_40_. In addition, no significant differences were detected in the expression of TNFa and IFNγ receptors. Neutrophils can attract cytotoxic T cells and activate dendritic cells through TNFα secretion [98]. Activated T cells secret IFNɣ that binds to its receptor IFNɣR1, stimulate neutrophils to support cytotoxic T cell activation, and enhance the tumor microenvironment’s responsiveness to immunotherapy [99]. The lack of changes in TNFα and IFNγ receptors suggests limited neutrophil involvement in these anti-tumor pathways.

It is worth noting that treatment with DNase I affected not only the phenotype of splenic neutrophils and the concentration of cfDNA, which correlated with previously obtained results [47], but also the histological features of the thymus and the number of macrophages in the tumor and thymus (Figure 3E,F and Figure 4A,C). The thymus is a central organ of immunogenesis, changes in which reflect the possible immunomodulatory effects of anti-tumor drugs. Histologically, the thymus of mice was represented by the cortex and medulla, the ratio of which differed between healthy and RLS_40_-bearing mice (Figure 4A). The thymus-cortex-to-medulla ratio increased in response to RLS_40_ tumor development; moreover, DNase I treatment enhanced this increase, providing evidence of the immunostimulatory properties of DNase I. It is very interesting that, even in samples where DNase I did not affect tumor development, an influx of macrophages into the tissue was noted. Interestingly, in the group treated with DNase, there was approximately the same number of monocytes and granulocytes as in the untreated control, although significantly more than in healthy animals. It is also worth noting that the attraction of macrophages to tumor tissue and thymus after treatment with DNase is combined with the observation that IL10 expression in neutrophils decreases to levels found in the spleens of healthy animals, since macrophages are considered the main source of the pan-leukocyte cytokine IL10. These observations highlight the multifunctionality and flexibility of neutrophil function in various settings.

*Proposed mechanisms of DNase I influence on tumor progression.* To elucidate the mechanisms of action of DNase I, the ratio of mitochondrial to nuclear DNA in the cfDNA pool was measured. As expected, DNase I caused a change in the mitochondrial/nuclear DNA ratio, with a significant reduction in the relative level of nuclear DNA and an increase in the level of mitochondrial DNA. Similar results were previously obtained in the analysis of cfDNA from BALF mice with acute pulmonary fibrosis treated intranasally with DNase I [64]. Interestingly, DNase I caused a change in the mitochondrial/nuclear cfDNA ratio in the blood only for RLS_40_, but in B16 mice, the changes were insignificant, which could be the cause or consequence of the subsequent different immune effects. Nevertheless, it is likely that the change in the mitochondrial/nuclear cfDNA ratio may be the trigger event, causing an additional influx of macrophages and a change in the neutrophil profile.

**Figure 7:**
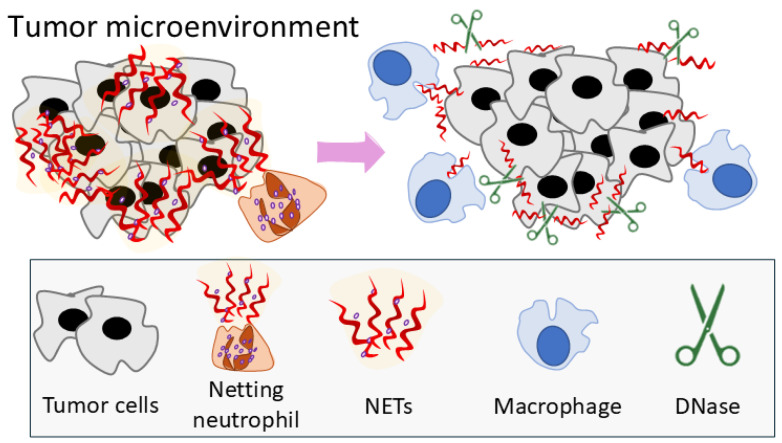
TANs and TAMs in the tumor microenvironment: proposed modulation of crosstalk following NETs degradation by DNase I.

The anti-tumor effects of DNase I could also be explained by the destruction of NETs (Figure 7).

NETs are known to protect tumor cells from cytotoxic T lymphocytes and interfere with the direct connection with the tumor cells in the TME and in circulation, which supports tumor growth and metastasis [54,100]. The destruction of NETs enables connections between cells in the TME. Apart from T cells, the macrophage population is a key player in the TME. The interaction between TANs and TAMs was shown to be critical in some cancers [101]. Moreover, macrophages have been shown to contribute in NET clearance [102]. NET clearance by macrophages could affect their phenotype and function. DNase I treatment destroys NETs and, in this way, could affect macrophage function (Figure 7). This may explain the observed macrophage infiltration in the tumor. In summary, the neutrophil phenotype corresponds with the tumor growth level, and DNase I treatment enhances the neutrophil anti-tumor characteristics and inhibits the immunosuppressive ones.

*Clinical relevance and existing clinical studies.* The tumor models employed in this study are highly relevant to human cancers. The LLC model closely parallels human non-small-cell lung cancer. The melanoma B16 model corresponds to human metastatic melanoma. The lymphosarcoma RLS_40_ model mimics human diffuse large B-cell lymphoma, characterized by multidrug resistance. Together, these models provide valuable platforms for studying cancer biology and therapies relevant to humans.

DNase I treatment has demonstrated promising anti-tumor and antimetastatic effects in this study, consistent with findings reported in previous research [42,43,44,45,46,47,48,49,50,51,52]. It is encouraging that DNase I—administered via various routes, including inhalation and intravenous infusion—has been and continues to be evaluated in multiple clinical trials (e.g., NCT02135588, NCT05453695, NCT02605590, NCT04541979, registered at https://clinicaltrials.gov/ (accessed 22 July 2025)). Although most of these trials focus on diseases other than cancer, they consistently demonstrate the low toxicity and favorable safety profile of DNase in humans.

## 5. Conclusions

The phenotype of neutrophils from tumor-bearing mice is influenced by the tumor type and progression stage. Neutrophils reflect the immune status of the organism and the immune response to the tumor. In the LLC model with aggressive progression, neutrophils adopt a general immunosuppressive phenotype, whereas, in the B16 model, due to its low immunogenicity, neutrophils show minimal phenotype alteration. However, in the RLS_40_ model, the phenotype of neutrophils was associated with the tumor growth rate, where, in neutrophils from mice with tumor progression, a sign of immunosuppressive phenotype was observed, while the neutrophils from mice with decreased tumor progression reflect an effective anti-tumor immunity. In the immunogenic model RLS_40_, DNase I had anti-tumor, antimetastatic, and immunostimulatory effects and significantly modified the neutrophil profile by decreasing the expression of the immunosuppressive markers (*Il10*, *Ccl17*) and increasing the expression of the anti-tumor markers (PD-L1, FAS). Moreover, DNase I treatment decreased the propensity of neutrophils for NETosis in response to chemical activation in vitro.

## Figures and Tables

**Table 1:** Concentration of blood serum cfDNA and mt/nucDNA ratio.

	cfDNA Concentration, ng/mL	mt DNA/nucDNA Ratio *
**RLS_40_ model**		
Healthy CBA mice	420 ± 120	0.1 ± 0.2
RLS_40_^High^	3100 ± 745 ##	1.0 ± 0.1 ##
RLS_40_^Med^	1711 ± 480 #	1.1 ± 0.1 ##
RLS_40_^Low^	1558 ± 668 #§	1.7 ± 0.1 ##§
RLS_40_^High^/D	2033 ± 947 ##	1.7 ± 0.1 ##
RLS_40_^Med^/D	822 ± 39 $†	2.1 ± 0.1 ##
RLS_40_^Low^/D	722 ± 394 §†	3.6 ± 0.2 ##§†
**B16 model**		
Healthy C57Bl mice	410 ± 120	0.1 ± 0.2
B16^High^	1320 ± 708 #	1.0 ± 0.1 ##
B16^Low^	806 ± 224	1.2 ± 0.2 ##
B16^High^/D	1577 ± 819 #	1.7 ± 0.3 ##
B16^Low^/D	872 ± 433	0.8 ± 0.07 ##

* mt DNA/nuc DNA ratio was calculated as relative levels of mitochondrial *Nd3* gene to nuclear *Actb* gene in cfDNA (qPCR data), #—statistical differences in comparison to healthy mice, §—differences between groups (RLS_40_^Low^ vs. RLS_40_^High^) or (B16^Low^ vs. B16^High^), Ψ—differences between groups (RLS_40_^Low^ vs. RLS_40_^Med^), $—differences between groups (RLS_40_^Med^ vs. RLS_40_^High^), †—differences between identical groups before and after DNase I treatment, ^single symbol^ *p* ≤ 0.05; ^double symbol^ *p* ≤ 0.01; ^triple symbol^ *p* ≤ 0.001; ^quadruple symbol^ *p* ≤ 0.0001.

## Data Availability

The raw data supporting the conclusions of this article will be made available by the corresponding author (N.L.M.) upon reasonable request.

## Supplementary Materials

The following supporting information can be downloaded at: https://www.mdpi.com/article/10.3390/cancers17162631/s1, Table S1. Primers and probes used in this study; Table S2. The antibodies used in the study; Table S3. RLS_40_-bearing mice distribution in subgroups treated with saline buffer (control) or DNase I; Table S4. Flow cytometry data showing % of cells expressing CD40, FAS, ICAM1, IFNGR1, and PD-L1 in the neutrophil gate (Ly6G^+^ events) from RLS_40_ groups; Table S5. Flow cytometry data showing % of cells expressing CD40, FAS, ICAM1, IFNGR1, and PD-L1 in the neutrophil gate (Ly6G^+^ events) from LLC group; Table S6. Flow cytometry data showing % of cells expressing CD40, FAS, ICAM1, IFNGR1, and PD-L1 in the neutrophil gate (Ly6G^+^ events) from B16 group; Table S7. RT-qPCR data for neutrophils of RLS_40_ groups: the expression level of corresponding gene normalized on *Tbp* and *Hprt1*; Table S8. RT-qPCR data for neutrophils of LLC groups: the expression level of corresponding gene normalized on *Tbp* and *Hprt1*; Table S9. RT-qPCR data for neutrophils of B16 groups: the expression level of the corresponding gene normalized on *Tbp* and *Hprt1*; Table S10. Neutrophil percentage in the spleen of tumor-bearing mice; Table S11. The effect of neutrophils, isolated from different RLS_40_-bearing mice subgroups, on RLS_40_ cells viability in vitro; Figure S1. Flow cytometry panel used to characterize splenic neutrophils. In the neutrophil gate (Ly6G^+^), the percent of neutrophils expressing PD-L1, FAS, ICAM-1, CD40, and IFNɣR1 were analyzed.
