# Supplementary material for: Neutrophils in Cancer: Phenotypic Heterogeneity Across Tumor Models and Significant Alteration of Splenic Neutrophil Phenotype in Lymphosarcoma RLS40 Model Following DNase I Treatment
Source: Cancers (Basel). 2025 Aug 12;17(16):2631. doi: 10.3390/cancers17162631 (PMC12384534; doi:10.3390/cancers17162631)
Supplement: Supplementary file 1 [file cancers-17-02631-s001.zip › cancers-3762059-supplementary.pdf]

Table S1. Primers and probes used in this study.

Gene	Sequences, 5′→3′		
	**Forward**	**Reverse**	**Probe**
Ccl17	CAGACCCCAAAGACAAACATG	GTCACAGGCCGTTTTATGTTG	FAM-TGACCTTCCCGCTGAGGCATT-BHQ1
Il10	AACATACTGCTAACCGACTCC	CAAATGCTCCTTGATTTCTGGG	FAM-ATCATTTCCGATAAGGCTTGGCAACC-BHQ1
Tnfa	TGGAGTCATTGCTCTGTGAAG	CCTGAGCCATAATCCCCTTTC	FAM-TCTGACCCCTTTACTCTGACCCCTT-BHQ1
Icam	GCAGAGGACCTTAACAGTCTAC	TACTTGGCTCCCTTCCGAGACCT	FAM-TACTTGGCTCCCTTCCGAGACCT-BHQ1
Mmp9	GACATAGACGGCATCCAGTATC	GTGGGAGGTATAGTGGGACA	FAM-TCGGCTGTGGTTCAGTTGTGGT-BHQ1
Vegfa	CCGAAACCATGAACTTTCTGC	CTTCATGGGACTTCTGCTCTC	FAM-CACTGGACCCTGGCTTTACTGCT-BHQ1
Cd274	CTCATTGTAGTGTCCACGGTC	ACGATCAGAGGGTTCAACAC	FAM- ACGCCACATTTCTCCACATCTAGCA-BHQ1
Vegfr1	AATGGCTTTCACGTTTCCTTG	CCGTAGCAGAATCCAGGTAATG	FAM- CCTCTCCTTCGGCTGGCATCTTT - BHQ1
Hgf	TGTGCCAACAGGTGTATCAG	CCAGTAGCATCGTTTTCTTGAC	FAM- TGCAAGTGAACGTAAAGCCCCTGT-BHQ1
Ern1	GCCCATCAACTTCCCTTCTAT	GACATCTTGTAGTCCACGTCG	FAM- TGCCACCTATTTTGACTATGCAGCCT - BHQ1
Fas	CTCAAGGTACTAATAGCATCTCCG	TCCTTGATATAATCCTTCTGAGCAG	FAM- TTATCAGTTTCACGAACCCGCCTCC - BHQ1
Sirt1	CTCTGAAAGTGAGACCAGTAGC	TGTAGATGAGGCAAAGGTTCC	FAM- TCCAAGTTCTATACCCCATGAAGTGCC - BHQ1
Stat3	GGCACCTTGGATTGAGAGTC	CGAAGGTTGTGCTGATAGAGG	FAM- AACGTGGCATGTGACTCTTTGCTG - BHQ1
Tbp	AAGAAAGGGAGAATCATGGACC	GAGTAAGTCCTGTGCCGTAAG	ROX-CCTGAGCATAAGGTGGAAGGCTGTT-BHQ2
Hprt1	CCCCAAAATGGTTAAGGTTGC	AACAAAGTCTGGCCTGTATCC	ROX-CTTGCTGGTGAAAAGGACCTCTCGAA-BHQ2
*Actb**	GAGGTATCCTGACCCTGAAGTA	TCTACAATGAGCTGCGTGTG	
*Nd3**	AATGCGGATTCGACCCTAC	CTTCTACTTCCACTACCATGAGC	

* primers for analysis of cfDNA content

Table S2. The antibodies used in the study.

	Studied marker	Fluorophore	Cat #
Neutrophil phenotype	Ly6G	violetFluor 450	AB253070
	CD54 (ICAM)	FITC	E-AB-F1018C
	CD119 (IFNγR1)	PE	E-AB-F1115D
	CD95 (Fas)	PE/Cy7	1363085
	CD40	PE/Dazzle™ 594	1223150
	PD-L1 (CD274)	APC	E-AB-F1132UE
Neutrophil percentage	CD45	FITC	553080
	CD11b	PerCP-Cy 5.5	550993
	Ly6G	PE	E-AB-F1108D

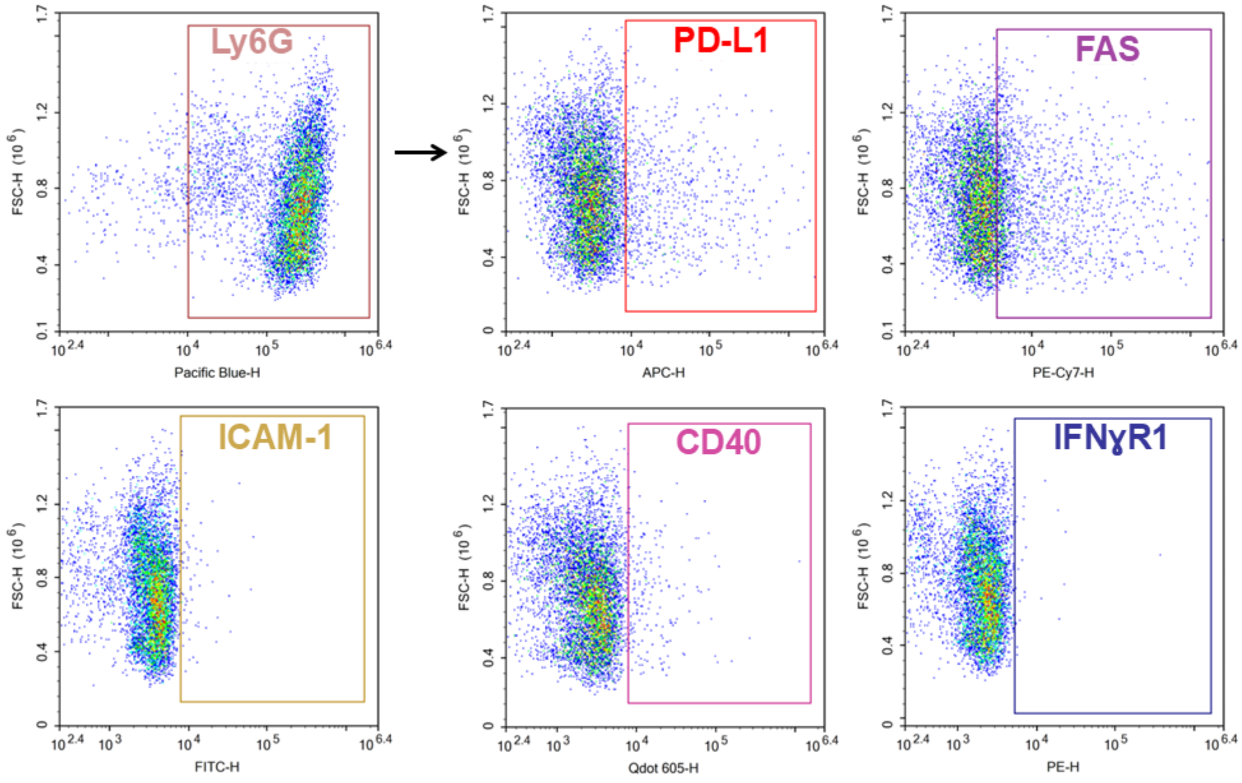

Figure S1. Flow cytometry panel used to characterize splenic neutrophils. In the neutrophil gate (Ly6G$^+$), the percent of neutrophils expressing PD-L1, FAS, ICAM-1, CD40 and IFNγR1 were analyzed.

Table S3. RLS_{40}-bearing mice distribution in subgroups treated with saline buffer (control) or DNase I.

	**Saline buffer**	**DNase I**
RLS_{40}^{High} (%/n)	(60.6 ± 1.7)/12	(44.5 ± 3.3)**/9
RLS_{40}^{Med} (%/n)	(14.1 ± 13.4)/3	(25.5 ± 7.3)/5
RLS_{40}^{Low} (%/n)	(25.3 ± 12.1)/5	(30.0 ± 4.0)/6

Data were analyzed using t test. ** $p < 0.01$.

Table S4. Flow cytometry data showing % of cells expressing CD40, FAS, ICAM1, IFNGR1 and PD-L1 in the neutrophil gate (Ly6G$^+$ events) from RLS_{40} groups.

	**HBM**	**HS**	**RLS_{40}^{Low}**	**RLS_{40}^{Low}/D**	**RLS_{40}^{Med}**	**RLS_{40}^{Med}/D**	**RLS_{40}^{High}**	**RLS_{40}^{High}/D**
CD40	0.3±0.1	0.6±0.1	2.1±0.01	2.3±0.1	2.4±0.02	1.9±0.3	0.3±0.01	0.4±0.1
ICAM1	0.5±0.04	0.±0.03	0.8±0.03	1.3±0.2	1.5±0.02	1.3±0.4	0.4±0.1	0.4±0.03
IFNGR1	0.2±0.02	0.4±0.1	0.3±0.01	2.0±0.4	2.3±0.0	1.6±0.6	0.2±0.01	0.2±0.01
FAS	0.3±0.03	0.7±0.1	20.6±1.9*	25.8±8.9*	1.4±0.1	5.6±1.5	2.8±0.3	1.9±0.8
PD-L1	0.3±0.05	0.4±0.1	7.8±0.02	20.0±4.1*	7.6±0.2	14.4±1.3	2.3±0.2	2.0±0.3

Results presented as mean±SEM. Data were statistically analyzed using non-parametric Kruskal-Wallis test with Dunn's multiple comparisons test and Bonferroni correction.

* Statistical differences in comparison to HBM;

# Differences in comparison to HS;

§ Differences between groups (RLS_{40}^{Low} vs RLS_{40}^{High});

Ψ Differences between groups (RLS_{40}^{Low} vs RLS_{40}^{Med});

$ Differences between groups (RLS_{40}^{Med} vs RLS_{40}^{High});

† Differences between identical groups before and after DNase I treatment;

$^{\text{single symbol}}$ $p \leq 0.05$; $^{\text{double symbol}}$ $p \leq 0.01$; $^{\text{triple symbol}}$ $p \leq 0.001$; $^{\text{quadruple symbol}}$ $p \leq 0.0001$.

Table S5. Flow cytometry data showing % of cells expressing CD40, FAS, ICAM1, IFNGR1 and PD-L1 in the neutrophil gate (Ly6G^{+} events) from LLC group.

	**HBM**	**HS**	**LLC**
CD40	0.3±0.1	0.3±0.02	1.0±0.1
ICAM1	0.4±0.1	0.5±0.03	2.00±0.9
IFNGR1	0.4±0.02	0.1±0.03	0.4±0.01
FAS	0.7±0.1	0.5±0.02	0.7±0.2
PD-L1	0.9±0.01	0.6±0.02	11.7±0.8#

Results presented as mean±SEM. Data were statistically analyzed non-parametric Kruskal-Wallis test with Dunn's multiple comparisons test and Bonferroni correction.
* Statistical differences in comparison to HBM;
# Differences in comparison to HS;
$^{\text{single symbol}}$ $p \leq 0.05$; $^{\text{double symbol}}$ $p \leq 0.01$; $^{\text{triple symbol}}$ $p \leq 0.001$; $^{\text{quadruple symbol}}$ $p \leq 0.0001$.

Table S6. Flow cytometry data showing % of cells expressing CD40, FAS, ICAM1, IFNGR1 and PD-L1 in the neutrophil gate (Ly6G$^+$ events) from B16 group.

	**HBM**	**HS**	**B16^{Low}**	**B16^{Low}/DNase**	**B16^{High}**	**B16^{High}/DNase**
CD40	0.3±0.1	0.3±0.02	6.8±0.1*, #	4.6±0.3	0. 8±0.1	1.4±0.01
ICAM1	0.4±0.1	0.5±0.03	5.4±0.04*	4.1±0.3	0.7±0.1	1.3±0.1
IFNGR1	0.4±0.02	0.1±0.03	4.9±0.2#	2.5±0.2	0.2±0.01	0.6±0.1
FAS	0.7±0.1	0.5±0.02	2.1±0.02	5.3±0.2##	0.6±0.02	1.7±0.2
PD-L1	0.9±0.01	0.6±0.02	3.4±0.1$$	3.2±0.04	0.5±0.01	0.9±0.1

Results presented as mean±SEM. Data were statistically analyzed using non-parametric Kruskal-Wallis test with Dunn's multiple comparisons test and Bonferroni correction.
* Statistical differences in comparison to HBM;
# Statistical differences in comparison to HS;
$ Statistical differences between groups (B16^{Low} vs B16^{High});
† Differences between identical groups before and after DNase I treatment;
$^{\text{single symbol}}$ $p \leq 0.05$; $^{\text{double symbol}}$ $p \leq 0.01$; $^{\text{triple symbol}}$ $p \leq 0.001$; $^{\text{quadruple symbol}}$ $p \leq 0.0001$.

Table S7. RT-qPCR data for neutrophils of RLS_{40} groups: the expression level of corresponding gene normalized on *Tbp* and *Hprt1*.

	**HBM**	**HS**	**RLS_{40}^{Low}**	**RLS_{40}^{Low}/D**	**RLS_{40}^{Med}**	**RLS_{40}^{Med}/D**	**RLS_{40}^{High}**	**RLS_{40}^{High}/D**
Ccl17	1.0±0.01	1.9±0.4	2.1±0.3	2.3±0.9	1.55±0.1	3.2±0.6	9.0±4.4	1.9±0.2
Il10	-	1.0±0.2	4.1±0.9*	1.6±0.2	4.2±0.01*	1.5±0.3	1.6±0.4	0.8±0.6
Icam1	1.0±0.1	3.8±2.5	9.9±2.1	15.1±2.8	4.1±0.1	48.1±0.6*,$	5.0±1.8	1.4±0.5
Tnfa	1.0±0.1	1.2±0.3	2.3±1.3	2.2±1.1	1.6±0.03	3.4±0.1	1.5±0.8	1.3±0.3
Cd274	1.0±0.2	4.3±0.5	25.0±4.3	81.8±12.5**	23.7±1.2	37.7±1.3	11.4±1.9	7.5±2.7
Fas	1.0±0.01	1.4±0.3	1.3±0.23	1.4±0.4	2.4±0.2	2.7±0.2	1.3±0.3	1.3±0.2
Sirt1	1.0±0.04	1.3±0.2	1.2±0.1	2.0±0.4	1.5±0.1	2.1±0.1	1.1±0.3	0.9±0.3
Hgf	1.1±0.2	0.5±0.1	0.4±0.3	1.1±0.5	0.6±0.01	1.4±0.4	0.7±0.1	0.6±0.2
Mmp9	1.0±0.1	0.2±0.1	0.3±0.1	0.8±0.4	0.2±0.02	2.00±0.1	0.2±0.1	0.2±0.1
Vegfa	1.0±0.1	1.9±0.2	1.2±0.6	1.9±0.5	1.9±0.1	3.2±0.1*	1.5±0.3	1.4±0.1
Vegfr1	1.0±0.2	5.9±0.1	7.2±2.1	6.1±2.9	7.1±0.5	12.9±5.0	2.6±0.6	4.2±0.8
Ern1	1.2±0.4	0.9±0.2	2.1±0.8	12.3±6.8	4.9±1.8	10.8±5.9	1.5±0.2	0.8±0.4
Stat3	1.1±0.3	0.5±0.1	0.7±0.5	8.1±4.4	0.7±0.1	5.5±3.0	0.7±0.2	0.6±0.03

Data were calculated using ΔΔCt method where samples of HBM were used as control, except for *Il10*, for which HS sample was used as a control. Results presented as Mean±SEM. Data were statistically analyzed using non-parametric Kruskal-Wallis test with Dunn's multiple comparisons test and Bonferroni correction.

* Statistical differences in comparison to HBM;

\# Differences in comparison to HS;

§ Differences between groups (RLS_{40}^{Low} vs RLS_{40}^{High});

Ψ Differences between groups (RLS_{40}^{Low} vs RLS_{40}^{Med});

$ Differences between groups (RLS_{40}^{Med} vs RLS_{40}^{High});

† Differences between identical groups before and after DNase I treatment;

$^{\text{single symbol}}$ $p \leq 0.05$; $^{\text{double symbol}}$ $p \leq 0.01$; $^{\text{triple symbol}}$ $p \leq 0.001$; $^{\text{quadruple symbol}}$ $p \leq 0.0001$.

.

Table S8. RT-qPCR data for neutrophils of LLC groups: the expression level of corresponding gene normalized on *Tbp* and *Hprt1*.

	**HBM**	**HS**	**LLC**
Ccl17	1.0±0.1	3.7±1.1	0.7±0.1#
Il10	1.0±0.5	4.1±1.2	78.4±25.2*
Icam1	1.0±0.2	0.3±0.1	26.6±17.0#
Tnfa	1.1±0.2	1.5±0.2	1.3±0.5
Cd274	1.1±0.3	1.3±0.2	23.3±16.3
Fas	1.0±0.1	1.2±0.2	0.7±0.3
Sirt1	1.0±0.2	0.9±0.1	1.0±0.2
Mmp9	1.0±0.4	0.1±0.02	0.8±0.7
Vegfa	1.0±0.1	1.7±0.8	0.5±0.2

Data were calculated using ΔΔCt method where samples of BM were used as control. Results presented as mean±SEM. Data were statistically analyzed non-parametric Kruskal-Wallis test with Dunn's multiple comparisons test and Bonferroni correction.

* Statistical differences in comparison to HBM;

# Differences in comparison to HS;

single symbol $p \leq 0.05$;

Table S9. RT-qPCR data for neutrophils of B16 groups: the expression level of corresponding gene normalized on *Tbp* and *Hprt1*.

	**HBM**	**HS**	**B16^{Low}**	**B16^{Low}/DNase**	**B16^{High}**	**B16^{High}/ DNase**
Icam1	1.1±0.3	0.9±0.5	0.2±0.0	0.5±0.1	1.4±0.6	1.8±0.5
Tnfa	1.0±0.1	0.6±0.2	0.2±0.0	0.4±0.1	0.6±0.04	1.0±0.2
Cd274	1.0±0.1	0.9±0.5	0.5±0.02	0.5±0.04	1.0±0.03	2.7±1.2
Fas	1.0±0.1	0.3±0.1	0.3±0.0	0.4±0.1	0.5±0.1	0.6±0.1
Sirt1	1.0±0.1	0.4±0.2	0.1±0.0*	0.3±0.1	0.38±0.02	0.7±0.1
Hgf	1.0±0.1	0.2±0.1	0.5±0.0	0.2±0.04	0.1±0.01	0.3±0.1
Mmp9	1.0±0.1	0.2±0.1	0.1±0.0	0.1±0.03	0.3±0.1	0.8±0.3
Vegfa	1.0±0.1	2.2±1.9	0.1±0.01	0.2±0.1	0.6±0.2	0.8±0.2
Ern1	1.1± 0.2	0.4±0.2	0.2±0.01	0.4±0.1	0.7±0.02	0.9±0.3
Stat3	1.2±0.5	0.1±0.1	0.2±0.0	0.4±0.2	0.3±0.1	1.3±0.4

Data are normalized on *Tbp* and *Hprt1* and calculated using ΔΔCt method where samples of BM were used as control. Results are presented as mean±SEM. Data were statistically analyzed using non-parametric Kruskal-Wallis test with Dunn's multiple comparisons test and Bonferroni correction.
* Statistical differences in comparison to HBM;
# Statistical differences in comparison to HS;
$ Statistical differences between groups (B16^{Low} vs B16^{High});
† Differences between identical groups before and after DNase I treatment;
$^{\text{single symbol}}$ $p \leq 0.05$; $^{\text{double symbol}}$ $p \leq 0.01$; $^{\text{triple symbol}}$ $p \leq 0.001$; $^{\text{quadruple symbol}}$ $p \leq 0.0001$.

Table S10. Neutrophil percentage in the spleen of tumor-bearing mice.

**Group**	**Neutrophil, %**
Healthy CBA (HS)	1.0±0.1
RLS_{40}^{Low}	1.9±0.2
RLS_{40}^{Med}	2.8±0.1
RLS_{40}^{High}	4.0±0.3#
RLS_{40}^{Low}/DNase	2.6±0.1
RLS_{40}^{Med}/DNase	2.2±0.1
RLS_{40}^{High} /DNase	3.3±0.8

Data are presented as mean ± SD. Data were analyzed using Kruskal–Wallis test with Dunn's multiple comparisons test and Bonferroni correction.
# Differences in comparison to HS;
§ Differences between groups (RLS_{40}^{Low} Vs RLS_{40}^{High});
Ψ Differences between groups (RLS_{40}^{Low} Vs RLS_{40}^{Med});
$ Differences between groups (RLS_{40}^{Med} Vs RLS_{40}^{High});
† Differences between identical groups before and after DNase I treatment;
$^{\text{single symbol}}$ $p \leq 0.05$; $^{\text{double symbol}}$ $p \leq 0.01$; $^{\text{triple symbol}}$ $p \leq 0.001$; $^{\text{quadruple symbol}}$ $p \leq 0.0001$.

Table S11. The effect of neutrophils, isolated from different RLS_{40}-bearing mice subgroups, on RLS_{40} cells viability *in vitro*.

	**RLS_{40} cell viability, %**
Control (without neutrophils)	80.7
RLS_{40}^{Low}	73.1
RLS_{40}^{Med}	74.4
RLS_{40}^{High}	73.1
RLS_{40}^{Low}/DNase	72.3
RLS_{40}^{Med}/DNase	73.7
RLS_{40}^{High}/DNase	68.9**

Data were analyzed using Kruskal–Wallis test with Dunn's multiple comparisons test and Bonferroni correction.
* Statistical differences in comparison to HBM;
# Differences in comparison to HS;
§ Differences between groups (RLS_{40}^{Low} Vs RLS_{40}^{High});
Ψ Differences between groups (RLS_{40}^{Low} Vs RLS_{40}^{Med});
$ Differences between groups (RLS_{40}^{Med} Vs RLS_{40}^{High});
† Differences between identical groups before and after DNase I treatment;
^single symbol^ $p \leq 0.05$; ^double symbol^ $p \leq 0.01$; ^triple symbol^ $p \leq 0.001$; ^quadruple symbol^ $p \leq 0.0001$.
